# Screening tools for employment in clinical healthcare delivery systems: a content analysis

**DOI:** 10.1186/s12913-024-10976-3

**Published:** 2024-06-11

**Authors:** Mina Colon, Julia M. Goodman

**Affiliations:** OHSU-PSU School of Public Health, Portland, OR USA

**Keywords:** Employment, Work, Social needs, Social risks, Social determinants, Screening, Survey, Healthcare services, Clinical healthcare delivery system

## Abstract

**Background:**

The relationship between work and health is complex and bidirectional, where work can have both health-harming and health-enhancing effects. Though employment is recognized as a social determinant of health, and clinical healthcare delivery systems are increasingly using screening tools to ask patients about social needs, little research has explored the extent to which employment-related social risk is captured in these screening tools. This study aimed to identify and characterize employment- and work-related questions in social risk screening tools that have been implemented in clinical healthcare delivery systems.

**Methods:**

We conducted a qualitative content analysis of employment-related items in screening tools that have been implemented in clinical healthcare service delivery systems. Three content areas guided data extraction and analysis: Setting, Domain, and Level of Contextualization.

**Results:**

Screening tools that asked employment-related questions were implemented in settings that were diverse in the populations served and the scope of care provided. The intent of employment-related items focused on four domains: Social Risk Factor, Social Need, Employment Exposure, and Legal Need. Most questions were found to have a low Level of Contextualization and were largely focused on identifying an individual’s employment status.

**Conclusions:**

Several existing screening tools include measures of employment-related social risk, but these items do not have a clear purpose and range widely depending on the setting in which they are implemented. In order to maximize the utility of these tools, clinical healthcare delivery systems should carefully consider what domain(s) they aim to capture and how they anticipate using the screening tools to address social determinants of health.

**Supplementary Information:**

The online version contains supplementary material available at 10.1186/s12913-024-10976-3.

## Background

Work is an important determinant of health inequities [1]. Work opportunities, and the corresponding risks and benefits, are strongly shaped by race, ethnicity, gender, age, class, and geography. Work influences where one fits in social and economic hierarchies, and is linked to education, income, and power [2–4].

The relationship between work and health is multidimensional and complex [2]. Work and health influence one another, and work can have both health-harming and health-enhancing effects [2, 3]. Work is a source of income and, in the United States, a key determinant of health insurance and access to healthcare [3]. As such, unemployment—a traditional measure of work, and precarious employment—relating to concepts like employment strain and employment uncertainty [5, 6], are important risk factors and have been linked to various adverse health outcomes [7, 8]. Work also determines exposure to environmental and occupational hazards, and is a source of psychological strain for many workers [9]. The complex ways work influences health have led to calls for using occupation or occupational prestige as an indicator of socioeconomic status beyond income and education [10].

Clinical healthcare delivery systems increasingly recognize the importance of identifying and addressing social determinants of health [9, 11–14], and conversations about screening for social risk factors have moved into the mainstream [14–16]. Screening patients within clinical healthcare delivery settings can provide data for practical applications on an individual level, like adapting medical care; a system level, like developing new healthcare models; and a societal level, like implementing population health interventions [17, 18]. Evidence gathered from screening tools creates opportunities to acutely improve patients’ social circumstances by providing a direct pathway to connect individuals to non-clinical services and provides pathways for long-term improvements [19]. The coordination of these services also supports integrated care models and generates insight into strategies to reduce healthcare spending [20].

According to the 2022 “State of the Science” report summarizing the state of social screening in healthcare, the estimated prevalence of social risk screening ranges from 56 to 77% [16]. However, a recent systematic review found that there were a limited number of studies that reported the impact of social risk screening and interventions on clinical outcomes like process measures, short-term social needs outcomes, intermediate impact on health outcomes, and long-term/health care cost or utilization outcomes [21]. Additionally, the measures and outcomes used in the studies are highly variable and underscore the difficulty in comparing and synthesizing the available data.

Despite the variability, available evidence, like that from Yan et al., supports the positive impact of integrating SDoH or social needs screening into electronic health records (EHRs) on the process, healthcare cost, and utilization measures and suggest positive associations with short-, intermediate-, and long-term health outcomes [21]. To further investigate the impact of screening and related interventions, such as referrals to non-clinical services to address social risk, it is integral to generate evidence that can directly test for the association between screening for social risk factors in clinical healthcare delivery settings and clinical health outcomes. In particular, there is a need to better understand how social risk screening impacts specific patient populations; how characteristics of single domains of social risk, like employment, can be leveraged to tailor screening tools for more effective patient care; and how the implementation of related interventions can influence health outcomes [14].

Employment is widely considered a social determinant of health [22], but is frequently included exclusively as a subset of economic stability [23]. For example, the State of the Science report combines employment and income as a single screening domain [16], and a systematic review of employment interventions in healthcare settings focused exclusively on interventions to help patients gain employment [24]. Neither of these summaries describes a focus on the characteristics of work or working conditions as a social risk factor. Thus, findings from this study will provide a comprehensive overview of the current landscape (setting, domain, and level of contextualization) of employment as a social risk screening item in clinical healthcare delivery settings. Evidence of setting will provide insight into the groups of patients being screened for employment; evidence of domain will generate a more nuanced understanding of the intent of screening for employment; and evidence of screening item contextualization will depict the variation in the depth with which social risk is screened for. Given the robust evidence linking employment status, specific working conditions, and occupational exposures to adverse health outcomes, this study sought to identify and characterize employment- and work-related questions in screening tools that have been evaluated in clinical healthcare delivery systems.

## Methods

We conducted a qualitative content analysis of employment-related items in screening tools that have been evaluated in clinical healthcare service delivery systems. An item is defined as the context of the question and its corresponding response options. A complete list of items (question and response options) can be found in ﻿Supplementary Table﻿ 1.

### Data sources

**Fig. 1:**
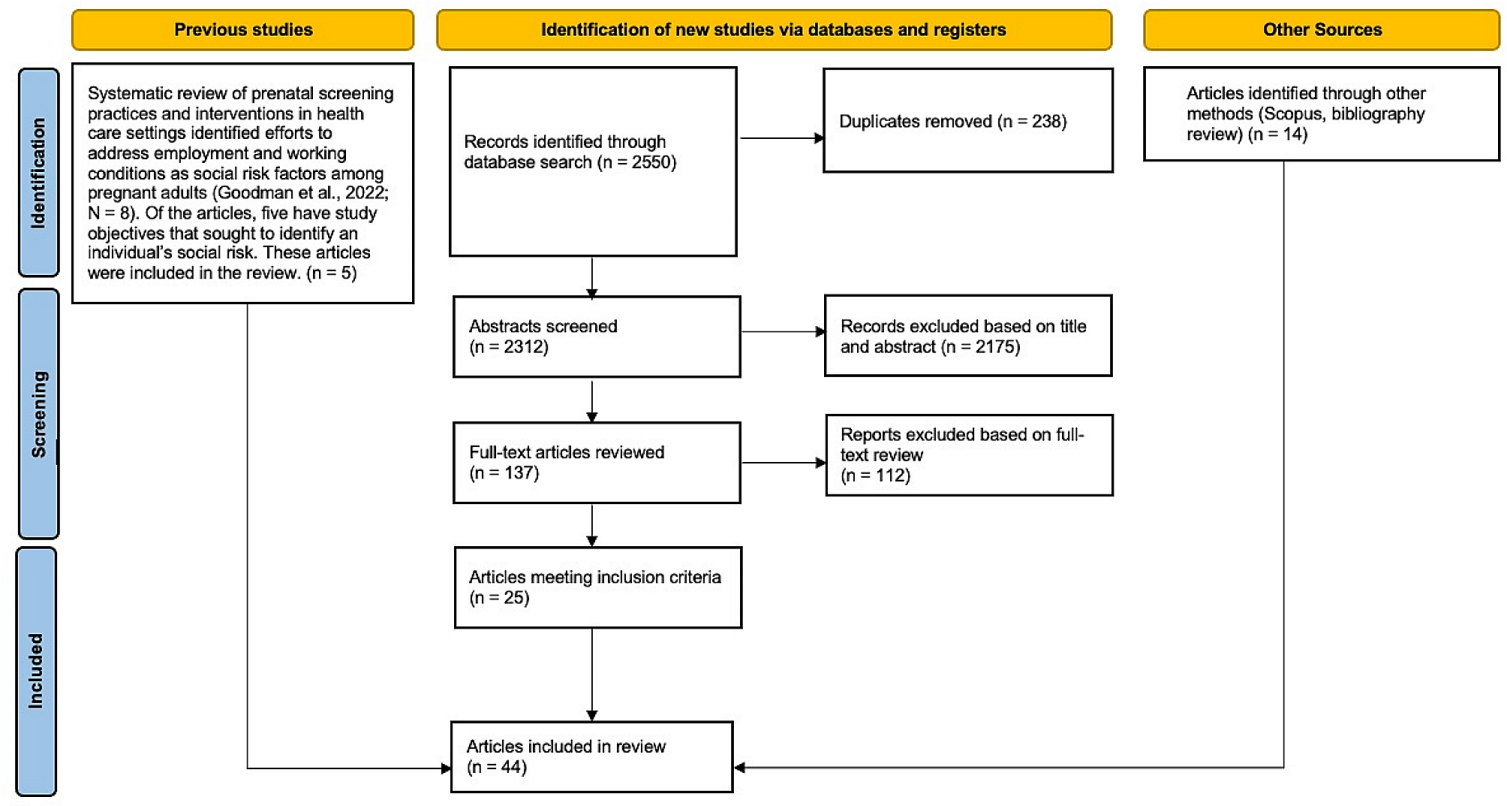
PRISMA flowchart. *Source* Page et al., 2021 [26]

The content analysis was based on literature from a related systematic review [25]. Using a comprehensive search strategy, we searched databases including MEDLINE, PsycINFO, SocINDEX, EMBASE, and the SIREN (Social Interventions and Research & Evaluation Network) Evidence and Resource library for studies that described screening tools for employment-related social risk factors published through February 14, 2022, as depicted in Fig. 1. In consultation with a reference librarian, we selected multiple search terms related to three domains: [1] employment or working conditions; [2] screening; and [3] healthcare settings. We supplemented our database search with hand searches of reference lists of included studies and by entering included studies into Scopus to identify citing articles. We also searched bibliographies of published systematic reviews that came up through our database search but excluded due to their study design. Our complete search strategy with all search terms is included in Supplementary File 1.

Studies were selected for the content analysis if [1] screening practices and/or interventions were integrated into a clinical healthcare service delivery system and [2] the screening tool assessed individuals for some employment exposure (e.g., employment, work, work arrangements, working conditions). Articles were excluded if they were published in languages other than English, were not empirical, were identified as a duplicate, screened patient populations outside of healthcare settings (e.g., in workplaces or other community settings), or used a screening tool that did not assess any dimension of employment.

Rayyan systematic review software was used to screen the article title and abstract. Two reviewers (J.G., M.C.) independently screened the articles. Discrepancies were solved by open discussion between reviewers. Articles selected for full-text review were then obtained and imported into EndNote, where they were independently assessed by the same two reviewers for inclusion. The final content analysis included 44 articles, describing 30 unique screening tools that included at least one employment-related item.

### Data extraction

Three content areas guided data extraction and analysis: Setting, Domain, and Level of Contextualization.

#### Setting

The setting was extracted from the article(s) describing each tool, and describes the clinical setting in which the screening tool was implemented. More than one setting listed next to a tool indicates that the tool was described in the context of multiple settings across the literature. Settings were diverse and included public health clinics, federally qualified health centers, hospitals, primary care practices, pediatric clinics, school-based clinics, community health centers, urgent care clinics, obstetric care facilities, and legal and homeless health clinics.

#### Domain

We characterized the primary intent of the question as the “domain.” We based our assessment on the objective(s) of the article in which the screening tool was described, the stated intent of the screening tool (if available), the screening tool sub-section in which the question was placed, and/or the language of the question itself. We identified four domains: Social Risk Factor, Social Need, Employment Exposure, and Legal Need. Questions in the Social Risk Factor domain sought to identify employment as an individual-level adverse social determinant of health—specifically, whether an individual lacked employment [27]. Those in the Social Need domain went beyond identifying employment status and included content that emphasized the patient’s role in prioritizing their needs and/or where its purpose within the screening tool was intended to provide a social intervention. The Employment Exposure domain captured questions that focus on occupational and environmental work exposures and/or working conditions, for example, prolonged standing or heavy lifting. Lastly, Legal Need asked about employment-related legal considerations.

#### Level of Contextualization

We characterized the Level of Contextualization based on the depth of inquiry into employment-related concerns and, correspondingly, the extent to which the screening tool could illuminate the context of the individual’s employment. We rated each set of items (per tool) Level 1–3, with higher Levels reflecting questions that capture more information about an individual’s employment context. Level 1 meant that the question asked whether an individual was employed or identified employment as a social need. Level 2 inquired about an individual’s type of employment or details about their work arrangement. This rating included responses that allowed individuals to identify the kind of work they engaged in (e.g., self-employed, homemaker) and details about their work arrangement (e.g., full-time, part-time, or temporary work). Level 3 was given for highly detailed content and in-depth questions about employment characteristics. These employment questions asked about specific workplace or occupational exposures and explored problems or barriers related to an individual’s particular work context.

## Results

We identified 30 unique screening tools that contained employment-related items (Table 1).

**Table 1:** Characteristics of screening tools with employment-related items

Screening Tool	Setting	Level of Cont.	Domain
Albright et al. [28]	• Veterans Affairs Medical Center, Federally Qualified Health Center, and Rural public health clinic	1	Social Risk Factor
Blue flags [29]	• Primary care centers	3	Employment Exposure
Cross-Sectional BRF survey [30, 31]	• Antenatal clinics at maternity hospitals • Antenatal clinics at hospitals	1	Social Risk Factor
Family fIRST [32]	• School-based pediatric clinic	1	Social Risk Factor
Fleeger et al. tool [33]	• Urban pediatric clinics	2	Social Need
Flinders University Social Health History Screening Tool (FUST) [34]	• Tertiary hospital	2	Social Need
Ganguli et al. tool [35]	• Primary Care	1	Social Need
Health Leads [36, 37]	• Hospital-based primary care practices • Internal medicine practices	1	Social Need
Health and Employment Resources: Opportunities for Success (HEROS) [38]	• Primary care centers	3	Employment Exposures
Ingleburn Baby Information System (IBIS) [39]	• South Western Sydney Area Health Service	2	Social Risk Factor
iScreen [40]	• Pediatric emergency department • Safety-net hospitals	1	Social Need
Mason et al. tool [41]	• Prenatal clinic	1	Social Risk Factor
Occupational Health Risk Assessment (OHRA) [42]	• Primary care clinic	3	Employment Exposures
Patient Reported Outcome Quality of Life Tool (PROQoL) [43, 44]	• Primary care practices • Family practice sites	1	Social Need
PRAPARE [45–49]	• Healthcare center clinic • Primary care federally qualified health center • Direct primary care • Community Health Center • Health centers	2	Social Need
Razani et al. tool [50]	• Federally Qualified Health Center and Urgent care clinic	2	Social Need
Reves et al. tool [51]	• General internal medicine inpatient services and Emergency department	1	Social Need
Schwartz et al. tool [52]	• Primary care clinic at a Veterans Affairs Medical Center	3	Employment Exposures
Semple-hess et al. tool [53]	• Urban children’s hospital	1	Social Need
Sokol et al. tool [54]	• Pediatric ambulatory care sites	1	Social Need
The Legal Health Check Up survey [55]	• Legal health clinic in an urban primary care setting	3	Legal Need
The Online Advocate [56]	• Adolescent and young adult medical practice	1	Social Need
THRIVE [57]	• Tertiary care medical center	1	Social Need
Tong et al. tool [58]	• Primary Care	2	Social Risk Factor
Tsai et al. survey [59]	• Homeless Health Clinics	1	Legal need
Van Beukering et al. tool [60]	• Obstetric care facilities	3	Employment Exposure
WE CARE [61–66]	• Urban hospital-based pediatric clinic • Urban community health centers • Urban community health centers • Two safety-net hospitals NICUs • Hospital-based pediatric clinic • Pediatric medical home clinic	1	Social Need
WellRX [67]	• Family medicine clinics	1	Social Need
Wiegner et al. tool [68]	• Primary Care clinics	1	Social Risk Factor
Zachek et al. tool [69]	• Women’s Health Center	3	Employment Exposure

### Setting

Screening tools with employment-related questions have been deployed in various healthcare service delivery systems (Table 1). Settings were diverse in the populations served and the scope of care provided. While many questions were asked in primary care settings, three notable populations of focus were identified in our content analysis as frequently appearing: pregnant, pediatric, and Veteran populations. The scope of care across settings also varied widely, and there was an observed spectrum of provision and subspecialty, with a range of examples including tertiary hospitals, school-based clinics, and community health centers.

### Domain

Questions about employment vary substantially and highlight the numerous ways employment is conceptualized in screening tools. The most common Domain that employment-related content addressed was Social Need (*n* = 15), with half of the screening tools assessing employment related to an individual’s prioritized needs or intending to provide a social intervention (Table 1). Seven screening tools included content assessing employment as a Social Risk Factor and six as an Employment Exposure. Two tools included content to assess employment as a Legal Need.

### Level of contextualization

Less than half of the items captured context beyond whether an individual is employed or needs employment. Most (*n* = 17) of the content assessed was rated a Level 1, primarily focusing on identifying an individual’s employment status. Item response options included binary (yes/no an individual is employed) and checkbox selections that asked participants to identify if employment was a need. Six of the items were rated a Level 2, with content related to the type of employment or work arrangements. Seven items were identified as having the most robust employment inquiry and were rated a Level 3. Items were detailed and included questions such as whether an individual has frequent noise or dermatologic exposures at their place of employment, or feels they have control over their work situation.

Six of the seven items rated a Level 3 were identified within the Employment Exposure domain (Table 2). Screening tool items that assess Social Risk or Social Need domains disproportionately lacked context, with most falling into Levels 1 and 2 of contextualization. In contrast, most items that assess Employment Exposure were highly contextualized (Level 3).

**Table 2:** Item level of contextualization by question domain

Level of context- ualization	Domain			
	Social risk factor	Social need	Employment exposure	Legal need
1.	• Albright et al. [28] • Cross-Sectional BRF survey [30, 31] • Family fIRST [32] • Mason et al. tool [41] • Wiegner et al. tool [68]	• Ganguli et al. tool [35] • Health Leads [36, 37] • iScreen [40] • Patient Reported Outcome Quality of Life Tool (PROQoL) [43, 44] • Reves et al. tool [51] • Semple-hess et al. tool [53] • Sokol et al. tool [54] • The Online Advocate [56] • THRIVE [57] • WE CARE [61–66] • WellRX [67]		• Tsai et al. survey [59]
2.	• Ingleburn Baby Information System (IBIS) [39] • Tong et al. tool [58]	• Fleeger et al. tool [33] • Flinders University Social Health History Screening Tool (FUST) [34] • PRAPARE [45–49] • Razani et al. tool [50]		
3.			• Blue flags [29] • Health and Employment Resources: Opportunities for Success (HEROS) [38] • Occupational Health Risk Assessment (OHRA) [42] • Schwartz et al. tool [52] • Van Beukering et al. tool [60] • Zachek et al. tool [69]	• The Legal Health Check Up survey [55]

## Discussion

Tools available to screen for employment in health systems are diverse in content and implementation, but relatively few assess the complex nature of work. Results from our analysis suggest that employment items in screening tools that have been evaluated in the included studies are underdeveloped and unclear in their intended purpose. In our content analysis, we first characterized the intent of the question, which we categorized into four Domains: Social Risk Factor, Social Need, Employment Exposure, and Legal Need. Half of the employment questions fell into the Social Need domain, which assessed respondents’ perceptions of employment as a concern, or which tied the question to an intervention. This Domain is similar to the Social Risk Factor domain, which assessed employment status in terms of whether the respondent was employed or what broad category of employment their work fell into (e.g., full-time vs. part-time work) without assessing the respondents’ need for support. In both domains, the purpose behind the question was unclear. If a respondent indicates that employment is a concern, does this suggest that they are concerned about finances, health insurance, or social support? Or, on the other hand, is their employment causing physical or psychological stress? Questions that fell into the Employment Exposure and Legal Need domains were clearer in their intent and more easily linked to a core measurement purpose (e.g., occupational exposure, environmental exposure, work characteristics, legal benefits, the legality of work arrangements).

Next, we examined the question and response options together and characterized the extent to which the item captures a respondent’s employment context. Together with the question Domain, this helps us understand how actionable a given item is. With more than half of the items assessed having little to no context (Level 1), our results suggest ambiguity in how screening tool questions are being developed, applied, and evaluated. Items without any employment context (e.g., “Are you employed? yes/no”) may stifle the potential benefits that screening tools can provide and have implications for how a healthcare delivery system can intervene to address the need. Even items that provided somewhat more employment context (Level 2) did not capture information specific enough for a provider to adjust one’s care plan or connect patients with resources [15]. For example, there was not enough information in these items glean details that would aid providers in discussing strategies to mitigate occupational risks based on an individual’s health status or help them make appropriate referrals to social services based on an individual’s need. In contrast, items identified as highly contextualized, with a Level 3, captured nuances that would allow providers to address specific aspects of an individual’s work context.

Of the tools identified that were highly contextualized, more than half were implemented in specialized healthcare delivery settings like musculoskeletal clinics and obstetric clinics. Providers in specialized settings may better understand the nuances of how employment status, working conditions, and occupational exposures impact their patients’ health. As such, providers in these settings are likely more incentivized to address employment in a contextual way to (1) better understand how best to treat a patient and (2) better identify measures of association between employment characteristics and health outcomes, which may be less apparent in primary care settings. Findings may also suggest that items of a tool reflect the level of impact the providers feel they have in modifying or addressing employment characteristics. For example, items in the Employment Exposure domain were mostly developed with an occupational health lens, while items in the Social Risk Factor and Social Need domains were more general. Occupational health providers may feel better equipped to understand how employment characteristics influence health and may feel more comfortable intervening to address a patient’s need. More exploratory evidence that looks at who developed the items, the primary intent, and the perceived modifiable risk would provide further insight into the relationship between contextualization and domain.

In addition to providing more concrete guidance for intervening, highly contextualized items with a clear purpose may encourage patient engagement if patients better understand how answering social screening questions are linked to solutions. While many patients believe that screening for social needs is valuable, patients need to be convinced that the screening tool items are intentional and to understand how providers will use the information [70, 71]. Future research should focus on developing items with a keener eye towards what context to include to adequately assess relevant outcomes of interest. Centering the intent of the item and considering the applicable contexts would provide a foundation for researchers to assess the effectiveness of individual questions more adequately. Implementation science frameworks, like the health equity implementation framework [72], would be particularly useful to describe how variation in question intent or clinical healthcare delivery setting might influence the effectiveness of screening tools given a particular setting or population.

Our analysis further examined the healthcare setting in which the screening tool has been used. Basic, less contextualized items might suffice for a general adult population where a wide range of social risks or social needs may be present, and the impact of work-related exposures could be less pronounced. However, our results suggest that specific populations may benefit from screening tool items better tailored to their needs. For example, pregnant, pediatric, and Veteran populations frequently appeared in our analysis, with each having different considerations and needs for care. Individuals in populations such as these could be at risk for more acute health implications warranting work adjustments or may more often experience working conditions that directly link to health. Distinguishing between settings is especially important in generating generalizable evidence across clinical healthcare delivery systems.

This study sought to identify and characterize employment- and work-related questions in screening tools implemented in clinical healthcare delivery systems. The observed variability in setting, domain, and level of contextualization suggests that it is challenging to assess the effectiveness of employment-related screening items and its direct impact on patient outcomes. The study had little insight into how individual screening tools were implemented and thus was limited in assessing the comparative effectiveness across the three content areas. Additionally, though patient health outcomes and clinical healthcare delivery system measures are critically important to capture in social needs interventions, like screening for employment, there is a lack of studies that assess the context of the screening tool and include common health and healthcare utilization outcomes. Therefore, there are few direct links that would allow this study to draw conclusions on the effectiveness of the screening tool on patient health and clinical healthcare delivery system outcomes.

Future research should consider the fragmented evidence available for interventions that bridge social and medical care. Assessing the setting, level of contextualization, and domains of screening tool items would provide other mechanisms to study comparative effectiveness and generate insight for providers to better tailor screening tools for patient care. The robust evidence connecting social determinants of health and health outcomes suggest that effectiveness research should explore outcomes beyond traditional measures of health and healthcare utilization. Including other measures of effectiveness such as short-term social needs, health-related behaviors, and quality of life could contribute to a better understanding of the impact of implementing screening tools.

## Conclusions

Clinical healthcare delivery systems have a substantial opportunity to adopt and leverage screening tool items to address social determinants of health. For a social factor, like employment, questions in screening tools must be clear about the purpose and consider the context—individual and setting—of implementation. In order to maximize the utility of these tools, clinical healthcare delivery systems should carefully consider why they are asking the question, who is being asked, how screening responses will help to address the need, and how success will be evaluated. Efforts to do so will influence the accuracy of identifying and assessing employment as a social determinant of health and provide a landscape for evaluative work to develop best practices.

### Electronic supplementary material

Below is the link to the electronic supplementary material.

Supplementary Material 1

Supplementary Material 2

## Data Availability

The dataset analyzed for the content analysis is available from the corresponding author. All literature included was obtained from publicly available sources.

## Abbreviations

SIREN: Social Interventions and Research & Evaluation Network
