# Supplementary material for: Screening tools for employment in clinical healthcare delivery systems: a content analysis
Source: BMC Health Serv Res. 2024 Jun 11;24:720. doi: 10.1186/s12913-024-10976-3 (PMC11167741; doi:10.1186/s12913-024-10976-3)
Supplement: Supplementary file 1 — Supplementary Material 1 [file 12913_2024_10976_MOESM1_ESM.docx]

Supplementary Table 1: Employment-related Item Content

The Setting, Level of Contextualization, and Domain characteristics according to employment-related items, including question and response contents assessed from each screening tool.

| **Screening Tool** | **Setting** | **Level of Cont.** | **Question** | **Response** | **Domain** |
| --- | --- | --- | --- | --- | --- |
| Albright et al [31] | - Veterans Affairs Medical Center, Federally Qualified Health Center, and Rural public health clinic | 1 | - Employment Status | - Employed or retire; unemployed; disabled | Social Risk Factor |
| Blue flags [32] | - Primary care centers | 3 | - My tasks at work are too difficult - There are incompatible demands for me at my work - I have too many tasks, too much work to do - I can count on that if necessary, get help and support from my colleagues - I can count on that if necessary, get help and support from my immediate supervisor - My work contains positive challenges - There are clear goals for my work - I can decide how fast I work - I have control in my work situation - Old and young staff are treated equally at my workplace - Men and women are treated equally at my workplace - I can solve problems that arise at work - There has been bullying and harassment at my workplace during the last 6 months - The work requires me to concentrate all the time and can make decisions | - Not relevant; relevant; very relevant | Employment Exposure |
| Cross-Sectional BRF survey [33,34] | - Antenatal clinics at maternity hospitals - Antenatal clinics at hospitals | 1 | - Is there at least one person in your household who currently has paid work/earns an income? - Have you had a job before? | - Yes/No | Social Risk Factor |
| Family fIRST [35] | - School-based pediatric clinic | 1 | - Are you currently working? | - Yes; No; Not sure | Social Risk Factor |
| Fleeger et al tool [36] | - Urban pediatric clinics | 2 | - What is your employment status? - Reason for difficulty finding/maintaining work - Use of job training/ job placement/interest in getting a paid job | - Employed; self-employed; out of work [duration]; homemaker; student; retired; unable to work - Open-ended | Social Need |
| Flinders University Social Health History Screening Tool  “FUST” [37] | - Tertiary hospital | 2 | - Are you working? - How many hours of paid work do you usually do per week? - How secure is your current job? | - Yes/No - Full time employed; Part time/ casual employment; Not Working; Home duties/retired; Student - Unable to (disability/work cover) - Very insecure (1) to insecure (5) | Social Need |
| Ganguli et al tool [38] | - Primary Care | 1 | - Are you experiencing financial worry? (Worrying about paying your bills, running out of food or affording food, or maintaining your income or job) | - Yes/No | Social Need |
| Health Leads [39,40] | - Hospital-based primary care practices - Internal medicine practices | 1 | - I would like help "finding employment or job training" | - Yes/No | Social Need |
| Health and Employment Resources: Opportunities for Success  HEROS [41] | - Primary care centers | 3 | - Employment status, job search self-efficacy, work performance, perceived employment barriers | - Scale ranging from 1 to 5, with higher scores indicating greater confidence in one’s ability to obtain employment | Employment Exposure |
| Ingleburn Baby Information System (IBIS) [42] | - South Western Sydney Area Health Service | 1 | - Difficulties finding or keeping a job | - Yes/No | Social Need |
| iScreen [43] | - Pediatric emergency department - Safety-net hospitals | 1 | - Is the patient employed? - Does that patient have problem coping with job? | - Yes/No | Social Risk Factor |
| Mason et al tool [44] | - Prenatal clinic | 3 | Dermatologic exposures   - In the past year have you worked with materials - that frequently made contact with your skin? In the past year have you had a skin rash you - feel was due to contact with any material? Respiratory exposures - Have you ever worked with any materials that were frequently present in the air you breathed? - Have any of these materials caused you breathing difficulty? (Including wheezing, shortness of breath, cough or phlegm)   Noise exposures   - Have you ever had a job where you had to frequently shout to be understood because of workplace noise? - Do you have difficulties with your hearing? (such - as ringing, deafness, or fluctuating hearing) Repetitive exposures - In your present job do you use any repetitive or awkward hand, arm, or shoulder positions for more than a few hours per day? - During the past 12 months have your work activities caused discomfort in your hand(s), arm(s), or shoulder(s) (including burning, stiff- ness, numbness, or tingling) lasting more than 1 week, or that has reoccurred more than once a month? - Are there any chemicals or substances in or around your home that you have concerns about? - Do you now or have you ever had any health problems that you feel are related to your job? - In your present job do you wear any protective equipment? (such as respirator, hearing protection, or gloves) | - Yes/No | Employment Exposure |
| Occupational Health Risk Assessment questionnaire (OHRA) [45] | - Primary care clinic | 1 | - Select top 3 priorities - Single biggest concern | - Personal relationships - Monitoring health Emotional health - Money - Health behaviors - Medicine - Getting healthcare - Work - Physical health - Something else? | Social Need |
| Patient Reported Outcome Quality of Life Tool (PROQoL) [46,47] | - Primary care practices - Family practice sites | 2 | - What is your current work situation? | - Unemployed; part-time or temporary work; full-time work; otherwise unemployed but not seeking work; chose not to answer/left blank | Social Need |
| PRAPARE [48-52] | - Healthcare center clinic - Primary care federally qualified health center - Direct primary care - Community Health Center - Health centers | 2 | - Trouble finding a job - Disability-related impairment interfering with ability to work - Problems with a current or former job, such as unpaid wages, worker’s compensation, wrongful termination, discrimination or harassment, or needing unemployment insurance - Concerns about pregnancy-related work benefits | - Yes/No | Social Need |
| Razani et al tool [53] | - Federally Qualified Health Center and Urgent care clinic | 1 | - Have you needed help within the last 30 days employment | - Yes/No | Social Need |
| Reves et al tool [54] | - General internal medicine inpatient services and Emergency department | 3 | - Employment status - Years employed in current job - Years employed in previous job - Health problem related to work - Do fellow works have similar health problems? - Occupation category - Occupational exposures | - Employed/retired - -Years-, -Years- - Yes/No - Yes/No - Yes/No - Professional Services; Laborer; Mechanic; Maintenance; Construction; Agriculture ; Transportation; Unknown - General; Chemical fumes; Solvents; Pesticides Heavy metals; Respiratory Asbestos; Coal dust; Silica; Grain dust Welding fumes; Physical Repetitive motion; Noise Radiation; Heat/cold | Employment Exposure |
| Schwartz et al tool [55] | - Primary care clinic at a Veterans Affairs Medical Center | 1 | - Select if: community resources in the past; anticipated community resource needs in the coming 12 months | - Public health clinics - Dental and/or vision care - Women’s health - Baby formula and/or breast-feeding support - Food banks, pantries, and/or meal services - Temporary housing and shelters Safe housing services Subsidized utilities Child and/or family therapy Parenting and/or - Parent support classes Parent education and/or - Job training Legal aid | Social Need |
| Semple-hess et al tool [56] | - Urban children's hospital | 1 | - In the last 4 weeks, have you been looking for work? - Do you need help finding a local career center and/or job training? | - Yes - No | Social Need |
| Sokol et al tool [57] | - Pediatric ambulatory care sites | 2 | - Father employment | - Fulltime, Casual/part time, Self emp, Unemployment, Other | Social Risk Factor |
| The Legal Health Check Up survey [58] | - Legal health clinic in an urban primary care setting | 3 | - Do you have a disability that affects your ability to work? Are you concerned about telling an employer about any health problems that you have? Have you ever been hurt at work? Is your workplace safe? Are you being harassed or discriminated against or being treated unfairly by your employer or a co-worker? Are you having trouble finding work because of the following (please check all that may apply):? Do you worry about getting fired, laid off, or having your hours cut? Do you have trouble getting time off when you need it to look after a family member? Do you need subsidized child care so you can work? Does your employer or past employer owe you money? Is there anything else you would like to tell us about employment issues? | - Yes - No | Legal Need |
| The Online Advocate [59] | - Adolescent and young adult medical practice | 1 | - Select any health-related social problem | - Exercise, nutrition, bodyweight - Education and after school programs Safety equipment use (car seats, helmets, smoke alarms) - Access to health care (medical, dental, insurance, prescriptions) Housing (availability, utilities, structural problems) - Food security - Income security ( job, income, education) - Substance use (tobacco, alcohol, drugs) - Sexual health - Violence (intimate partner and violence risk factors) | Social Need |
| THRIVE [60] | - Tertiary care medical center | 1 | - Are you currently unemployed and looking for a job? | - Yes/No | Social Need |
| Tong et al tool [61] | - Primary Care | 2 | - What is your current source of income? | - Employed full time; Employed part time; Homemaker; Retired; Unemployed; Disabled; Social Security/Government assistance; Student; Retired; Other; I choose not to answer | Social Risk Factor |
| Tsai et al survey [62] | - Homeless Health Clinics | 1 | - Which civil legal issues were most commonly encountered and which most affected patient health. | - Employment; Health insurance; Medicaid enrollment | Legal need |
| Van Beukering et al tool [63] | - Obstetric care facilities | 3 | Work: General aspects   - Paid work? - Working in sector? - Number of employees in the company - Travel distance commuting (m/km) - Travel time commuting - Means of travelling/transport   Current Work Status   - Information from employer when reporting pregnancy - Information about advice to adjust work from? From who? - Working times - Irregular working times - Physical work - Physical work: regularly/ often - Job strain: often/always - Exposed to biological agents - Exposed to chemical agents | - Yes from the start of pregnancy - Yes from X week of pregnancy - Health care; Business services and research; Education; Welfare and child care; Retail & Hotel and catering industry; Government & Culture; Recreation; Other (Industry/NGO’s/ transport) - # of employees: 1-10, 11-50, 51-100, more than 100 - <5 km; 5-10 km; 10-25 km; >25 km - <1 hour/day; 1-2 hours/day; >2 hours/day - Walking, by bicycle/scooter, public transport, car   Current Work Status   - Normal Working hours; Part time due to illness; No work due to illness; Pregnancy leave - Work adjustments; Pregnancy/maternity leave   - Midwife and obstetrician   - Occupational physician   - Manager or staff advisor   - Own initiative - Less physically demanding; Less standing or walking; Fewer hours/day; More working from home   - Hours/week   - Days/week - Evening shifts; Night shifts - Standing/walking ≥4 hours/day; Lifting/carrying loads or people - Bending; Physically very demanding; Requiring physical strength - Problems with pressure; Like to take things a little easier; Freedom in performance of tasks; Influence on the pace to work; Planning own work; Support from manager; Enjoy working; Finds work satisfying - Yes/No - Yes/No | Employment Exposure |
| WE CARE [64-69] | - Urban hospital-based pediatric clinic - Urban community health centers - Urban community health centers - Two safety-net hospitals NICUs - Hospital-based pediatric clinic - Pediatric medical home clinic | 1 | - Do you have a job? | - Yes/No | Social Need |
| WellRX [70] | - Family medicine clinics | 1 | - Are you unemployed or without regular income? - Do you need help finding a better job? | - Yes/No | Social Need |
| Wiegner et al tool [71] | - Primary Care clinics | 1 | - What is your occupational status? | - Employed; Unemployed; Other | Social Risk Factor |
| Zachek et al tool [72] | - Women’s Health Center | 3 | - How many jobs have you had since you became pregnant? ______ - What kind of businesses or industries have you worked at since you became pregnant? - What is name or title of your job or jobs? - What kind of work or activities do you do at the jobs you have had since you became pregnant? - How many hours per week do you work at these jobs? - Since you became pregnant, have you worked in any of these businesses or industries? - Since you became pregnant, have you come into contact with any chemicals at your job? - Since you became pregnant, have you smelled chemicals at your job? - Do you know where to find the Material Safety Data Sheets for chemicals that are used at your job? - Since you became pregnant, how often have you used these chemicals at your job? - Since you became pregnant, have you had any of these symptoms at your job? - Do you think these symptoms are linked to your work - environment? - Do these symptoms go away when you are home or away from your job? - Do the people you work with have any of these symptoms? - Do you use any of these safety tools at your job? - Are you concerned that your job is harming your health? | - Open ended - Open ended - Open ended - Open ended - Janitor or house cleaning, gas station, hair salon, construction, nail salon, healthcare, dry cleaning, dentist, car or truck repair, science lab, farm, electronic manufacturing, plant nursery, semiconductor manufacturing - Yard work, hazardous waste, printing company, plastics recycling, chemical plant, other factory - Every day; some days; never - Clean floors, counters, sinks, toilers; lift/push/pull heavy things; stand for a long time; use/make/handle pesticide; work with glues or adhesives; welding; degrease tools/machines/electronics; X-ray/CT/radiotherapy/nuclear medicine; mix or apply paints; remove or strip paint - Yes; No; Don’t know - Yes; No; Don’t know - Yes; No; Doesn’t apply - Every day; some days; never   - Janitorial chemicals   - Solvents   - Dry cleaning   - Paint   - Nail polish remover   - Paint stripper   - Pesticides   - Lead   - Glues/adhesive   - Mercury   - Degreasers   - Lab Chemicals   - Ethylene Oxide   - X-rays   - Nitrous oxide   - Radioactive materials   - Anesthesia gases   - Other chemical or metal - Yes/No   - Headache   - Dizziness   - Itchy or teary eyes   - Nausea   - Sneezing or bloody nose   - Vomiting   - Coughing or sore throat   - Other symptom   - Hives, rash, or itchy skin - Yes; No; I don’t have symptoms - Yes; No; I don’t have symptoms - Yes; No; Don’t know; I don’t have symptoms - Yes/No   - Gloves   - Lab hood   - Protective clothing   - Air vents/fans   - Respirators or masks   - Other tools - Yes; No | Employment Exposure |
