# Supplementary material for: Screening tools for employment in clinical healthcare delivery systems: a content analysis
Source: BMC Health Serv Res. 2024 Jun 11;24:720. doi: 10.1186/s12913-024-10976-3 (PMC11167741; doi:10.1186/s12913-024-10976-3)
Supplement: Supplementary file 2 — Supplementary Material 2 [file 12913_2024_10976_MOESM2_ESM.docx]

**[1]**

**Database: MEDLINE**

**Platform: Ovid**

**Date of Search: 1/12/21**

**# of Retrieved Results: 272**

**Search Strategy:**

1 ((screen* or survey* or question* or ask*) adj7 ((employ* or unemploy* or job or jobs or work or working or occupation* or career*) adj3 (status* or situation* or condition* or histor*))).mp. [mp=title, abstract, original title, name of substance word, subject heading word, floating sub-heading word, keyword heading word, organism supplementary concept word, protocol supplementary concept word, rare disease supplementary concept word, unique identifier, synonyms] (2806)

2 exp Mass Screening/ (130663)

3 exp Employment/ (88505)

4 2 and 3 (841)

5 AAFP Social Needs Screening Tool.mp. [mp=title, abstract, original title, name of substance word, subject heading word, floating sub-heading word, keyword heading word, organism supplementary concept word, protocol supplementary concept word, rare disease supplementary concept word, unique identifier, synonyms] (0)

6 Social Needs Screening Tool*.mp. [mp=title, abstract, original title, name of substance word, subject heading word, floating sub-heading word, keyword heading word, organism supplementary concept word, protocol supplementary concept word, rare disease supplementary concept word, unique identifier, synonyms] (3)

7 AccessHealth Spartanburg Screening Tool.mp. [mp=title, abstract, original title, name of substance word, subject heading word, floating sub-heading word, keyword heading word, organism supplementary concept word, protocol supplementary concept word, rare disease supplementary concept word, unique identifier, synonyms] (0)

8 ((AccessHealth or Spartanburg) adj3 Screening Tool*).mp. [mp=title, abstract, original title, name of substance word, subject heading word, floating sub-heading word, keyword heading word, organism supplementary concept word, protocol supplementary concept word, rare disease supplementary concept word, unique identifier, synonyms] (0)

9 Accountable Health Communities Health-Related Social Needs Screening Tool.mp. [mp=title, abstract, original title, name of substance word, subject heading word, floating sub-heading word, keyword heading word, organism supplementary concept word, protocol supplementary concept word, rare disease supplementary concept word, unique identifier, synonyms] (0)

10 Boston Medical Center-Thrive Screening Tool.mp. [mp=title, abstract, original title, name of substance word, subject heading word, floating sub-heading word, keyword heading word, organism supplementary concept word, protocol supplementary concept word, rare disease supplementary concept word, unique identifier, synonyms] (0)

11 HealthBegins Upstream Risk Screening Tool.mp. [mp=title, abstract, original title, name of substance word, subject heading word, floating sub-heading word, keyword heading word, organism supplementary concept word, protocol supplementary concept word, rare disease supplementary concept word, unique identifier, synonyms] (0)

12 Medical-Legal Partnership IHELLP.mp. [mp=title, abstract, original title, name of substance word, subject heading word, floating sub-heading word, keyword heading word, organism supplementary concept word, protocol supplementary concept word, rare disease supplementary concept word, unique identifier, synonyms] (0)

13 PRAPARE.mp. [mp=title, abstract, original title, name of substance word, subject heading word, floating sub-heading word, keyword heading word, organism supplementary concept word, protocol supplementary concept word, rare disease supplementary concept word, unique identifier, synonyms] (5)

14 WellRx Toolkit.mp. [mp=title, abstract, original title, name of substance word, subject heading word, floating sub-heading word, keyword heading word, organism supplementary concept word, protocol supplementary concept word, rare disease supplementary concept word, unique identifier, synonyms] (0)

15 We Care Screening Tool.mp. [mp=title, abstract, original title, name of substance word, subject heading word, floating sub-heading word, keyword heading word, organism supplementary concept word, protocol supplementary concept word, rare disease supplementary concept word, unique identifier, synonyms] (1)

16 iscreen.mp. [mp=title, abstract, original title, name of substance word, subject heading word, floating sub-heading word, keyword heading word, organism supplementary concept word, protocol supplementary concept word, rare disease supplementary concept word, unique identifier, synonyms] (13)

17 Family fIRST.mp. [mp=title, abstract, original title, name of substance word, subject heading word, floating sub-heading word, keyword heading word, organism supplementary concept word, protocol supplementary concept word, rare disease supplementary concept word, unique identifier, synonyms] (181)

18 screen*.mp. [mp=title, abstract, original title, name of substance word, subject heading word, floating sub-heading word, keyword heading word, organism supplementary concept word, protocol supplementary concept word, rare disease supplementary concept word, unique identifier, synonyms] (861468)

19 17 and 18 (9)

20 ((social* or psychosoc*) adj5 (determin* or risk*) adj7 (employment* or employed or employee* or unemploy* or job or jobs or occupation*)).mp. [mp=title, abstract, original title, name of substance word, subject heading word, floating sub-heading word, keyword heading word, organism supplementary concept word, protocol supplementary concept word, rare disease supplementary concept word, unique identifier, synonyms] (836)

21 exp "Social Determinants of Health"/ (3715)

22 ((social* or psychosoc*) adj2 (determin* or risk*) adj5 (health* or unhealth*)).mp. [mp=title, abstract, original title, name of substance word, subject heading word, floating sub-heading word, keyword heading word, organism supplementary concept word, protocol supplementary concept word, rare disease supplementary concept word, unique identifier, synonyms] (9880)

23 21 or 22 (9880)

24 exp Socioeconomic Factors/ (458860)

25 (employ* or unemploy* or job or jobs or work or worked or works or working).mp. [mp=title, abstract, original title, name of substance word, subject heading word, floating sub-heading word, keyword heading word, organism supplementary concept word, protocol supplementary concept word, rare disease supplementary concept word, unique identifier, synonyms] (1972009)

26 23 or 24 (465507)

27 2 and 25 and 26 (1333)

28 ((social* or psychosoc*) adj5 (determin* or risk*) adj7 (employ* or unemploy* or job or jobs or work or worked or works or working)).mp. (1452)

29 ((social* or psychosoc*) adj5 (determin* or risk*) adj7 ((employ* or unemploy* or job or jobs or work or worked or works or working) adj5 (question* or screen* or tool* or survey* or ask* or answer*))).mp. (49)

30 exp Data Collection/ (2238696)

31 exp Health Status/ (350965)

32 26 or 31 (768586)

33 30 and 32 (311923)

34 3 and 33 (25911)

35 2 and 34 (558)

36 (question* or screen* or tool* or survey* or ask* or answer*).mp. [mp=title, abstract, original title, name of substance word, subject heading word, floating sub-heading word, keyword heading word, organism supplementary concept word, protocol supplementary concept word, rare disease supplementary concept word, unique identifier, synonyms] (3170834)

37 28 and 36 (761)

38 1 or 4 or 29 or 35 or 37 (4368)

39 exp General Practice/ (75448)

40 general practitioners/ or physicians, family/ or physicians, primary care/ (28069)

41 exp Primary Health Care/ (164166)

42 39 or 40 or 41 (249266)

43 ((family adj3 (medic* or care or healthcare or practic*)) or (general adj3 (medic* or care or healthcare or practic*)) or (primary adj3 (care or healthcare))).mp. [mp=title, abstract, original title, name of substance word, subject heading word, floating sub-heading word, keyword heading word, organism supplementary concept word, protocol supplementary concept word, rare disease supplementary concept word, unique identifier, synonyms] (312561)

44 42 or 43 (400963)

45 38 and 44 (205)

46 5 or 6 or 7 or 8 or 9 or 10 or 11 or 12 or 13 or 14 or 15 or 16 or 17 (203)

47 44 and 46 (10)

48 45 or 47 (215)

49 20 or 23 (10525)

50 2 or 36 (3178549)

51 44 and 49 and 50 (440)

52 51 not 48 (425)

53 4 and 33 (558)

54 3 and 23 and 30 (147)

55 53 or 54 (704)

56 55 not (48 or 51) (660)

57 exp "Delivery of Health Care"/ (1102533)

58 56 and 57 (141)

59 exp risk/ (1240239)

60 56 and 59 (171)

61 58 or 60 (272)

***************************

**[2]**

**Database: MEDLINE**

**Platform: Ovid**

**Date of Search: 1/12/21**

**# of Retrieved Results: 215**

**Search Strategy:**

1 ((screen* or survey* or question* or ask*) adj7 ((employ* or unemploy* or job or jobs or work or working or occupation* or career*) adj3 (status* or situation* or condition* or histor*))).mp. [mp=title, abstract, original title, name of substance word, subject heading word, floating sub-heading word, keyword heading word, organism supplementary concept word, protocol supplementary concept word, rare disease supplementary concept word, unique identifier, synonyms] (2805)

2 exp Mass Screening/ (130662)

3 exp Employment/ (88503)

4 2 and 3 (841)

5 AAFP Social Needs Screening Tool.mp. [mp=title, abstract, original title, name of substance word, subject heading word, floating sub-heading word, keyword heading word, organism supplementary concept word, protocol supplementary concept word, rare disease supplementary concept word, unique identifier, synonyms] (0)

6 Social Needs Screening Tool*.mp. [mp=title, abstract, original title, name of substance word, subject heading word, floating sub-heading word, keyword heading word, organism supplementary concept word, protocol supplementary concept word, rare disease supplementary concept word, unique identifier, synonyms] (3)

7 AccessHealth Spartanburg Screening Tool.mp. [mp=title, abstract, original title, name of substance word, subject heading word, floating sub-heading word, keyword heading word, organism supplementary concept word, protocol supplementary concept word, rare disease supplementary concept word, unique identifier, synonyms] (0)

8 ((AccessHealth or Spartanburg) adj3 Screening Tool*).mp. [mp=title, abstract, original title, name of substance word, subject heading word, floating sub-heading word, keyword heading word, organism supplementary concept word, protocol supplementary concept word, rare disease supplementary concept word, unique identifier, synonyms] (0)

9 Accountable Health Communities Health-Related Social Needs Screening Tool.mp. [mp=title, abstract, original title, name of substance word, subject heading word, floating sub-heading word, keyword heading word, organism supplementary concept word, protocol supplementary concept word, rare disease supplementary concept word, unique identifier, synonyms] (0)

10 Boston Medical Center-Thrive Screening Tool.mp. [mp=title, abstract, original title, name of substance word, subject heading word, floating sub-heading word, keyword heading word, organism supplementary concept word, protocol supplementary concept word, rare disease supplementary concept word, unique identifier, synonyms] (0)

11 HealthBegins Upstream Risk Screening Tool.mp. [mp=title, abstract, original title, name of substance word, subject heading word, floating sub-heading word, keyword heading word, organism supplementary concept word, protocol supplementary concept word, rare disease supplementary concept word, unique identifier, synonyms] (0)

12 Medical-Legal Partnership IHELLP.mp. [mp=title, abstract, original title, name of substance word, subject heading word, floating sub-heading word, keyword heading word, organism supplementary concept word, protocol supplementary concept word, rare disease supplementary concept word, unique identifier, synonyms] (0)

13 PRAPARE.mp. [mp=title, abstract, original title, name of substance word, subject heading word, floating sub-heading word, keyword heading word, organism supplementary concept word, protocol supplementary concept word, rare disease supplementary concept word, unique identifier, synonyms] (5)

14 WellRx Toolkit.mp. [mp=title, abstract, original title, name of substance word, subject heading word, floating sub-heading word, keyword heading word, organism supplementary concept word, protocol supplementary concept word, rare disease supplementary concept word, unique identifier, synonyms] (0)

15 We Care Screening Tool.mp. [mp=title, abstract, original title, name of substance word, subject heading word, floating sub-heading word, keyword heading word, organism supplementary concept word, protocol supplementary concept word, rare disease supplementary concept word, unique identifier, synonyms] (1)

16 iscreen.mp. [mp=title, abstract, original title, name of substance word, subject heading word, floating sub-heading word, keyword heading word, organism supplementary concept word, protocol supplementary concept word, rare disease supplementary concept word, unique identifier, synonyms] (13)

17 Family fIRST.mp. [mp=title, abstract, original title, name of substance word, subject heading word, floating sub-heading word, keyword heading word, organism supplementary concept word, protocol supplementary concept word, rare disease supplementary concept word, unique identifier, synonyms] (181)

18 screen*.mp. [mp=title, abstract, original title, name of substance word, subject heading word, floating sub-heading word, keyword heading word, organism supplementary concept word, protocol supplementary concept word, rare disease supplementary concept word, unique identifier, synonyms] (861312)

19 17 and 18 (9)

20 ((social* or psychosoc*) adj5 (determin* or risk*) adj7 (employment* or employed or employee* or unemploy* or job or jobs or occupation*)).mp. [mp=title, abstract, original title, name of substance word, subject heading word, floating sub-heading word, keyword heading word, organism supplementary concept word, protocol supplementary concept word, rare disease supplementary concept word, unique identifier, synonyms] (835)

21 exp "Social Determinants of Health"/ (3715)

22 ((social* or psychosoc*) adj2 (determin* or risk*) adj5 (health* or unhealth*)).mp. [mp=title, abstract, original title, name of substance word, subject heading word, floating sub-heading word, keyword heading word, organism supplementary concept word, protocol supplementary concept word, rare disease supplementary concept word, unique identifier, synonyms] (9880)

23 21 or 22 (9880)

24 exp Socioeconomic Factors/ (458858)

25 (employ* or unemploy* or job or jobs or work or worked or works or working).mp. [mp=title, abstract, original title, name of substance word, subject heading word, floating sub-heading word, keyword heading word, organism supplementary concept word, protocol supplementary concept word, rare disease supplementary concept word, unique identifier, synonyms] (1971730)

26 23 or 24 (465505)

27 2 and 25 and 26 (1333)

28 ((social* or psychosoc*) adj5 (determin* or risk*) adj7 (employ* or unemploy* or job or jobs or work or worked or works or working)).mp. (1452)

29 ((social* or psychosoc*) adj5 (determin* or risk*) adj7 ((employ* or unemploy* or job or jobs or work or worked or works or working) adj5 (question* or screen* or tool* or survey* or ask* or answer*))).mp. (49)

30 exp Data Collection/ (2238658)

31 exp Health Status/ (350962)

32 26 or 31 (768581)

33 30 and 32 (311921)

34 3 and 33 (25911)

35 2 and 34 (558)

36 (question* or screen* or tool* or survey* or ask* or answer*).mp. [mp=title, abstract, original title, name of substance word, subject heading word, floating sub-heading word, keyword heading word, organism supplementary concept word, protocol supplementary concept word, rare disease supplementary concept word, unique identifier, synonyms] (3170246)

37 28 and 36 (761)

38 1 or 4 or 29 or 35 or 37 (4367)

39 exp General Practice/ (75448)

40 general practitioners/ or physicians, family/ or physicians, primary care/ (28068)

41 exp Primary Health Care/ (164162)

42 39 or 40 or 41 (249261)

43 ((family adj3 (medic* or care or healthcare or practic*)) or (general adj3 (medic* or care or healthcare or practic*)) or (primary adj3 (care or healthcare))).mp. [mp=title, abstract, original title, name of substance word, subject heading word, floating sub-heading word, keyword heading word, organism supplementary concept word, protocol supplementary concept word, rare disease supplementary concept word, unique identifier, synonyms] (312526)

44 42 or 43 (400925)

45 38 and 44 (205)

46 5 or 6 or 7 or 8 or 9 or 10 or 11 or 12 or 13 or 14 or 15 or 16 or 17 (203)

47 44 and 46 (10)

48 45 or 47 (215)

49 20 or 23 (10524)

50 2 or 36 (3177961)

51 44 and 49 and 50 (440)

52 51 not 48 (425)

***************************

**[3]**

**Database: MEDLINE**

**Platform: Ovid**

**Date of Search: 1/12/21**

**# of Retrieved Results: 425**

**Search Strategy:**

1 ((screen* or survey* or question* or ask*) adj7 ((employ* or unemploy* or job or jobs or work or working or occupation* or career*) adj3 (status* or situation* or condition* or histor*))).mp. [mp=title, abstract, original title, name of substance word, subject heading word, floating sub-heading word, keyword heading word, organism supplementary concept word, protocol supplementary concept word, rare disease supplementary concept word, unique identifier, synonyms] (2805)

2 exp Mass Screening/ (130662)

3 exp Employment/ (88503)

4 2 and 3 (841)

5 AAFP Social Needs Screening Tool.mp. [mp=title, abstract, original title, name of substance word, subject heading word, floating sub-heading word, keyword heading word, organism supplementary concept word, protocol supplementary concept word, rare disease supplementary concept word, unique identifier, synonyms] (0)

6 Social Needs Screening Tool*.mp. [mp=title, abstract, original title, name of substance word, subject heading word, floating sub-heading word, keyword heading word, organism supplementary concept word, protocol supplementary concept word, rare disease supplementary concept word, unique identifier, synonyms] (3)

7 AccessHealth Spartanburg Screening Tool.mp. [mp=title, abstract, original title, name of substance word, subject heading word, floating sub-heading word, keyword heading word, organism supplementary concept word, protocol supplementary concept word, rare disease supplementary concept word, unique identifier, synonyms] (0)

8 ((AccessHealth or Spartanburg) adj3 Screening Tool*).mp. [mp=title, abstract, original title, name of substance word, subject heading word, floating sub-heading word, keyword heading word, organism supplementary concept word, protocol supplementary concept word, rare disease supplementary concept word, unique identifier, synonyms] (0)

9 Accountable Health Communities Health-Related Social Needs Screening Tool.mp. [mp=title, abstract, original title, name of substance word, subject heading word, floating sub-heading word, keyword heading word, organism supplementary concept word, protocol supplementary concept word, rare disease supplementary concept word, unique identifier, synonyms] (0)

10 Boston Medical Center-Thrive Screening Tool.mp. [mp=title, abstract, original title, name of substance word, subject heading word, floating sub-heading word, keyword heading word, organism supplementary concept word, protocol supplementary concept word, rare disease supplementary concept word, unique identifier, synonyms] (0)

11 HealthBegins Upstream Risk Screening Tool.mp. [mp=title, abstract, original title, name of substance word, subject heading word, floating sub-heading word, keyword heading word, organism supplementary concept word, protocol supplementary concept word, rare disease supplementary concept word, unique identifier, synonyms] (0)

12 Medical-Legal Partnership IHELLP.mp. [mp=title, abstract, original title, name of substance word, subject heading word, floating sub-heading word, keyword heading word, organism supplementary concept word, protocol supplementary concept word, rare disease supplementary concept word, unique identifier, synonyms] (0)

13 PRAPARE.mp. [mp=title, abstract, original title, name of substance word, subject heading word, floating sub-heading word, keyword heading word, organism supplementary concept word, protocol supplementary concept word, rare disease supplementary concept word, unique identifier, synonyms] (5)

14 WellRx Toolkit.mp. [mp=title, abstract, original title, name of substance word, subject heading word, floating sub-heading word, keyword heading word, organism supplementary concept word, protocol supplementary concept word, rare disease supplementary concept word, unique identifier, synonyms] (0)

15 We Care Screening Tool.mp. [mp=title, abstract, original title, name of substance word, subject heading word, floating sub-heading word, keyword heading word, organism supplementary concept word, protocol supplementary concept word, rare disease supplementary concept word, unique identifier, synonyms] (1)

16 iscreen.mp. [mp=title, abstract, original title, name of substance word, subject heading word, floating sub-heading word, keyword heading word, organism supplementary concept word, protocol supplementary concept word, rare disease supplementary concept word, unique identifier, synonyms] (13)

17 Family fIRST.mp. [mp=title, abstract, original title, name of substance word, subject heading word, floating sub-heading word, keyword heading word, organism supplementary concept word, protocol supplementary concept word, rare disease supplementary concept word, unique identifier, synonyms] (181)

18 screen*.mp. [mp=title, abstract, original title, name of substance word, subject heading word, floating sub-heading word, keyword heading word, organism supplementary concept word, protocol supplementary concept word, rare disease supplementary concept word, unique identifier, synonyms] (861312)

19 17 and 18 (9)

20 ((social* or psychosoc*) adj5 (determin* or risk*) adj7 (employment* or employed or employee* or unemploy* or job or jobs or occupation*)).mp. [mp=title, abstract, original title, name of substance word, subject heading word, floating sub-heading word, keyword heading word, organism supplementary concept word, protocol supplementary concept word, rare disease supplementary concept word, unique identifier, synonyms] (835)

21 exp "Social Determinants of Health"/ (3715)

22 ((social* or psychosoc*) adj2 (determin* or risk*) adj5 (health* or unhealth*)).mp. [mp=title, abstract, original title, name of substance word, subject heading word, floating sub-heading word, keyword heading word, organism supplementary concept word, protocol supplementary concept word, rare disease supplementary concept word, unique identifier, synonyms] (9880)

23 21 or 22 (9880)

24 exp Socioeconomic Factors/ (458858)

25 (employ* or unemploy* or job or jobs or work or worked or works or working).mp. [mp=title, abstract, original title, name of substance word, subject heading word, floating sub-heading word, keyword heading word, organism supplementary concept word, protocol supplementary concept word, rare disease supplementary concept word, unique identifier, synonyms] (1971730)

26 23 or 24 (465505)

27 2 and 25 and 26 (1333)

28 ((social* or psychosoc*) adj5 (determin* or risk*) adj7 (employ* or unemploy* or job or jobs or work or worked or works or working)).mp. (1452)

29 ((social* or psychosoc*) adj5 (determin* or risk*) adj7 ((employ* or unemploy* or job or jobs or work or worked or works or working) adj5 (question* or screen* or tool* or survey* or ask* or answer*))).mp. (49)

30 exp Data Collection/ (2238658)

31 exp Health Status/ (350962)

32 26 or 31 (768581)

33 30 and 32 (311921)

34 3 and 33 (25911)

35 2 and 34 (558)

36 (question* or screen* or tool* or survey* or ask* or answer*).mp. [mp=title, abstract, original title, name of substance word, subject heading word, floating sub-heading word, keyword heading word, organism supplementary concept word, protocol supplementary concept word, rare disease supplementary concept word, unique identifier, synonyms] (3170246)

37 28 and 36 (761)

38 1 or 4 or 29 or 35 or 37 (4367)

39 exp General Practice/ (75448)

40 general practitioners/ or physicians, family/ or physicians, primary care/ (28068)

41 exp Primary Health Care/ (164162)

42 39 or 40 or 41 (249261)

43 ((family adj3 (medic* or care or healthcare or practic*)) or (general adj3 (medic* or care or healthcare or practic*)) or (primary adj3 (care or healthcare))).mp. [mp=title, abstract, original title, name of substance word, subject heading word, floating sub-heading word, keyword heading word, organism supplementary concept word, protocol supplementary concept word, rare disease supplementary concept word, unique identifier, synonyms] (312526)

44 42 or 43 (400925)

45 38 and 44 (205)

46 5 or 6 or 7 or 8 or 9 or 10 or 11 or 12 or 13 or 14 or 15 or 16 or 17 (203)

47 44 and 46 (10)

48 45 or 47 (215)

49 20 or 23 (10524)

50 2 or 36 (3177961)

51 44 and 49 and 50 (440)

52 51 not 48 (425)

***************************

**[4]**

**Database: Embase**

**Platform: Embase.com**

**Date of Search: 4/22/21**

**# of Retrieved Results: 133**

**Search Strategy:**

((('mass screening'/exp AND (((screen* OR survey* OR question* OR ask*) NEAR/7 (employ* OR unemploy* OR job OR jobs OR work OR working OR occupation* OR career*) OR ('mass screening'/exp AND 'employment'/exp) OR ((social* OR psychosoc*) NEAR/5 (determin* OR risk*) AND (determin* OR risk*) NEAR/7 (employ* OR unemploy* OR job OR jobs OR work OR worked OR works OR working) AND (employ* OR unemploy* OR job OR jobs OR work OR worked OR works OR working) NEAR/5 (question* OR screen* OR tool* OR survey* OR ask* OR answer*)) OR ('mass screening'/exp AND ('employment'/exp AND ('data collection method'/exp AND ((('social determinants of health'/exp OR ((social* OR psychosoc*) NEAR/2 (determin* OR risk*) AND (determin* OR risk*) NEAR/5 (health* OR unhealth*))) OR 'socioeconomics'/exp) OR ('health status'/exp OR 'health status indicator'/exp))))) OR (((social* OR psychosoc*) NEAR/5 (determin* OR risk*) AND (determin* OR risk*) NEAR/7 (employ* OR unemploy* OR job OR jobs OR work OR worked OR works OR working)) AND (question*:ti,ab,de,tn OR screen*:ti,ab,de,tn OR tool*:ti,ab,de,tn OR survey*:ti,ab,de,tn OR ask*:ti,ab,de,tn OR answer*:ti,ab,de,tn))) AND (('pregnancy'/exp OR 'pregnancy complication'/exp OR 'maternal care'/exp OR 'maternal exposure'/exp OR 'obstetrics'/exp OR 'obstetric procedure'/exp) OR (pregnant* OR obstetric* OR maternal* OR perinat* OR prenat*)))) AND ('socioeconomics'/exp AND 'data collection method'/exp AND (((screen* OR survey* OR question* OR ask*) NEAR/7 (employ* OR unemploy* OR job OR jobs OR work OR working OR occupation* OR career*) OR ('mass screening'/exp AND 'employment'/exp) OR ((social* OR psychosoc*) NEAR/5 (determin* OR risk*) AND (determin* OR risk*) NEAR/7 (employ* OR unemploy* OR job OR jobs OR work OR worked OR works OR working) AND (employ* OR unemploy* OR job OR jobs OR work OR worked OR works OR working) NEAR/5 (question* OR screen* OR tool* OR survey* OR ask* OR answer*)) OR ('mass screening'/exp AND ('employment'/exp AND ('data collection method'/exp AND ((('social determinants of health'/exp OR ((social* OR psychosoc*) NEAR/2 (determin* OR risk*) AND (determin* OR risk*) NEAR/5 (health* OR unhealth*))) OR 'socioeconomics'/exp) OR ('health status'/exp OR 'health status indicator'/exp))))) OR (((social* OR psychosoc*) NEAR/5 (determin* OR risk*) AND (determin* OR risk*) NEAR/7 (employ* OR unemploy* OR job OR jobs OR work OR worked OR works OR working)) AND (question*:ti,ab,de,tn OR screen*:ti,ab,de,tn OR tool*:ti,ab,de,tn OR survey*:ti,ab,de,tn OR ask*:ti,ab,de,tn OR answer*:ti,ab,de,tn))) AND (('pregnancy'/exp OR 'pregnancy complication'/exp OR 'maternal care'/exp OR 'maternal exposure'/exp OR 'obstetrics'/exp OR 'obstetric procedure'/exp) OR (pregnant* OR obstetric* OR maternal* OR perinat* OR prenat*))))) OR (('mass screening'/exp AND (((screen* OR survey* OR question* OR ask*) NEAR/7 (employ* OR unemploy* OR job OR jobs OR work OR working OR occupation* OR career*) OR ('mass screening'/exp AND 'employment'/exp) OR ((social* OR psychosoc*) NEAR/5 (determin* OR risk*) AND (determin* OR risk*) NEAR/7 (employ* OR unemploy* OR job OR jobs OR work OR worked OR works OR working) AND (employ* OR unemploy* OR job OR jobs OR work OR worked OR works OR working) NEAR/5 (question* OR screen* OR tool* OR survey* OR ask* OR answer*)) OR ('mass screening'/exp AND ('employment'/exp AND ('data collection method'/exp AND ((('social determinants of health'/exp OR ((social* OR psychosoc*) NEAR/2 (determin* OR risk*) AND (determin* OR risk*) NEAR/5 (health* OR unhealth*))) OR 'socioeconomics'/exp) OR ('health status'/exp OR 'health status indicator'/exp))))) OR (((social* OR psychosoc*) NEAR/5 (determin* OR risk*) AND (determin* OR risk*) NEAR/7 (employ* OR unemploy* OR job OR jobs OR work OR worked OR works OR working)) AND (question*:ti,ab,de,tn OR screen*:ti,ab,de,tn OR tool*:ti,ab,de,tn OR survey*:ti,ab,de,tn OR ask*:ti,ab,de,tn OR answer*:ti,ab,de,tn))) AND (('pregnancy'/exp OR 'pregnancy complication'/exp OR 'maternal care'/exp OR 'maternal exposure'/exp OR 'obstetrics'/exp OR 'obstetric procedure'/exp) OR (pregnant* OR obstetric* OR maternal* OR perinat* OR prenat*)))) AND ('employment'/exp AND (((screen* OR survey* OR question* OR ask*) NEAR/7 (employ* OR unemploy* OR job OR jobs OR work OR working OR occupation* OR career*) OR ('mass screening'/exp AND 'employment'/exp) OR ((social* OR psychosoc*) NEAR/5 (determin* OR risk*) AND (determin* OR risk*) NEAR/7 (employ* OR unemploy* OR job OR jobs OR work OR worked OR works OR working) AND (employ* OR unemploy* OR job OR jobs OR work OR worked OR works OR working) NEAR/5 (question* OR screen* OR tool* OR survey* OR ask* OR answer*)) OR ('mass screening'/exp AND ('employment'/exp AND ('data collection method'/exp AND ((('social determinants of health'/exp OR ((social* OR psychosoc*) NEAR/2 (determin* OR risk*) AND (determin* OR risk*) NEAR/5 (health* OR unhealth*))) OR 'socioeconomics'/exp) OR ('health status'/exp OR 'health status indicator'/exp))))) OR (((social* OR psychosoc*) NEAR/5 (determin* OR risk*) AND (determin* OR risk*) NEAR/7 (employ* OR unemploy* OR job OR jobs OR work OR worked OR works OR working)) AND (question*:ti,ab,de,tn OR screen*:ti,ab,de,tn OR tool*:ti,ab,de,tn OR survey*:ti,ab,de,tn OR ask*:ti,ab,de,tn OR answer*:ti,ab,de,tn))) AND (('pregnancy'/exp OR 'pregnancy complication'/exp OR 'maternal care'/exp OR 'maternal exposure'/exp OR 'obstetrics'/exp OR 'obstetric procedure'/exp) OR (pregnant* OR obstetric* OR maternal* OR perinat* OR prenat*))))) OR (('socioeconomics'/exp AND 'data collection method'/exp AND (((screen* OR survey* OR question* OR ask*) NEAR/7 (employ* OR unemploy* OR job OR jobs OR work OR working OR occupation* OR career*) OR ('mass screening'/exp AND 'employment'/exp) OR ((social* OR psychosoc*) NEAR/5 (determin* OR risk*) AND (determin* OR risk*) NEAR/7 (employ* OR unemploy* OR job OR jobs OR work OR worked OR works OR working) AND (employ* OR unemploy* OR job OR jobs OR work OR worked OR works OR working) NEAR/5 (question* OR screen* OR tool* OR survey* OR ask* OR answer*)) OR ('mass screening'/exp AND ('employment'/exp AND ('data collection method'/exp AND ((('social determinants of health'/exp OR ((social* OR psychosoc*) NEAR/2 (determin* OR risk*) AND (determin* OR risk*) NEAR/5 (health* OR unhealth*))) OR 'socioeconomics'/exp) OR ('health status'/exp OR 'health status indicator'/exp))))) OR (((social* OR psychosoc*) NEAR/5 (determin* OR risk*) AND (determin* OR risk*) NEAR/7 (employ* OR unemploy* OR job OR jobs OR work OR worked OR works OR working)) AND (question*:ti,ab,de,tn OR screen*:ti,ab,de,tn OR tool*:ti,ab,de,tn OR survey*:ti,ab,de,tn OR ask*:ti,ab,de,tn OR answer*:ti,ab,de,tn))) AND (('pregnancy'/exp OR 'pregnancy complication'/exp OR 'maternal care'/exp OR 'maternal exposure'/exp OR 'obstetrics'/exp OR 'obstetric procedure'/exp) OR (pregnant* OR obstetric* OR maternal* OR perinat* OR prenat*)))) AND ('employment'/exp AND (((screen* OR survey* OR question* OR ask*) NEAR/7 (employ* OR unemploy* OR job OR jobs OR work OR working OR occupation* OR career*) OR ('mass screening'/exp AND 'employment'/exp) OR ((social* OR psychosoc*) NEAR/5 (determin* OR risk*) AND (determin* OR risk*) NEAR/7 (employ* OR unemploy* OR job OR jobs OR work OR worked OR works OR working) AND (employ* OR unemploy* OR job OR jobs OR work OR worked OR works OR working) NEAR/5 (question* OR screen* OR tool* OR survey* OR ask* OR answer*)) OR ('mass screening'/exp AND ('employment'/exp AND ('data collection method'/exp AND ((('social determinants of health'/exp OR ((social* OR psychosoc*) NEAR/2 (determin* OR risk*) AND (determin* OR risk*) NEAR/5 (health* OR unhealth*))) OR 'socioeconomics'/exp) OR ('health status'/exp OR 'health status indicator'/exp))))) OR (((social* OR psychosoc*) NEAR/5 (determin* OR risk*) AND (determin* OR risk*) NEAR/7 (employ* OR unemploy* OR job OR jobs OR work OR worked OR works OR working)) AND (question*:ti,ab,de,tn OR screen*:ti,ab,de,tn OR tool*:ti,ab,de,tn OR survey*:ti,ab,de,tn OR ask*:ti,ab,de,tn OR answer*:ti,ab,de,tn))) AND (('pregnancy'/exp OR 'pregnancy complication'/exp OR 'maternal care'/exp OR 'maternal exposure'/exp OR 'obstetrics'/exp OR 'obstetric procedure'/exp) OR (pregnant* OR obstetric* OR maternal* OR perinat* OR prenat*)))))) AND [embase]/lim

-------------------------------------

**[5]**

**Database: SocINDEX**

**Platform: EBSCO**

**Date of Search: 4/22/21**

**# of Retrieved Results: 338**

**Search Strategy:**

Search ID# Search Terms Search Options Last Run Via Results

S34 S27 AND S33 Expanders - Apply equivalent subjects

Search modes - Boolean/Phrase Interface - EBSCOhost Research Databases

Search Screen - Advanced Search

Database - SocINDEX with Full Text 338

S33 S31 OR S32 Expanders - Apply equivalent subjects

Search modes - Boolean/Phrase Interface - EBSCOhost Research Databases

Search Screen - Advanced Search

Database - SocINDEX with Full Text 44,890

S32 (pregnan* OR obstet* OR childbear* OR perinatal* OR peri-natal* OR

prenatal* OR pre-natal* OR (family N2 planning) OR ((maternal* OR

mother*) N3 (health* OR welfar* OR expos* OR risk*))) Expanders - Apply

equivalent subjects

Search modes - Boolean/Phrase Interface - EBSCOhost Research Databases

Search Screen - Advanced Search

Database - SocINDEX with Full Text 43,730

S31 S28 OR S29 OR S30 Expanders - Apply equivalent subjects

Search modes - Boolean/Phrase Interface - EBSCOhost Research Databases

Search Screen - Advanced Search

Database - SocINDEX with Full Text 12,308

S30 DE "OBSTETRICS" OR DE "CHILDBIRTH" OR DE "MIDWIFERY" Expanders -

Apply equivalent subjects

Search modes - Boolean/Phrase Interface - EBSCOhost Research Databases

Search Screen - Advanced Search

Database - SocINDEX with Full Text 4,247

S29 DE "MATERNAL health" Expanders - Apply equivalent subjects

Search modes - Boolean/Phrase Interface - EBSCOhost Research Databases

Search Screen - Advanced Search

Database - SocINDEX with Full Text 491

S28 DE "PREGNANCY" OR DE "EXTRAMARITAL pregnancy" OR DE "MALE

pregnancy" OR DE "MULTIPLE pregnancy" OR DE "PARITY (Obstetrics)" OR DE

"PREGNANT women" OR DE "TEENAGE pregnancy" OR DE "UNPLANNED pregnancy"

OR DE "UNWANTED pregnancy" Expanders - Apply equivalent subjects

Search modes - Boolean/Phrase Interface - EBSCOhost Research Databases

Search Screen - Advanced Search

Database - SocINDEX with Full Text 8,709

S27 S1 OR S4 OR S24 OR S26 Expanders - Apply equivalent subjects

Search modes - Boolean/Phrase Interface - EBSCOhost Research Databases

Search Screen - Advanced Search

Database - SocINDEX with Full Text 10,168

S26 S17 AND S25 Expanders - Apply equivalent subjects

Search modes - Boolean/Phrase Interface - EBSCOhost Research Databases

Search Screen - Advanced Search

Database - SocINDEX with Full Text 378

S25 (question* OR screen* OR tool* OR survey* OR ask* OR answer*)

Expanders - Apply equivalent subjects

Search modes - Boolean/Phrase Interface - EBSCOhost Research Databases

Search Screen - Advanced Search

Database - SocINDEX with Full Text 413,160

S24 S16 OR S18 OR S23 Expanders - Apply equivalent subjects

Search modes - Boolean/Phrase Interface - EBSCOhost Research Databases

Search Screen - Advanced Search

Database - SocINDEX with Full Text 76

S23 S3 AND S22 Expanders - Apply equivalent subjects

Search modes - Boolean/Phrase Interface - EBSCOhost Research Databases

Search Screen - Advanced Search

Database - SocINDEX with Full Text 20

S22 S20 AND S21 Expanders - Apply equivalent subjects

Search modes - Boolean/Phrase Interface - EBSCOhost Research Databases

Search Screen - Advanced Search

Database - SocINDEX with Full Text 800

S21 DE "SOCIAL science research" OR DE "ACTION research" OR DE

"COMMUNITY life research" OR DE "COMMUNITY-based participatory research"

OR DE "ETHNOSTATISTICS" OR DE "EVALUATION research (Social action

programs)" OR DE "ORGANIZATIONAL research" OR DE "PSYCHOLOGICAL

research" OR DE "SOCIAL network analysis" OR DE "SOCIAL surveys" OR DE

"SOCIOLOGICAL imagination" OR DE "SOCIOLOGICAL research" OR DE

"STATISTICS on social sciences" OR DE "TELEPHONE surveys" Expanders -

Apply equivalent subjects

Search modes - Boolean/Phrase Interface - EBSCOhost Research Databases

Search Screen - Advanced Search

Database - SocINDEX with Full Text 27,991

S20 S14 OR S19 Expanders - Apply equivalent subjects

Search modes - Boolean/Phrase Interface - EBSCOhost Research Databases

Search Screen - Advanced Search

Database - SocINDEX with Full Text 73,365

S19 DE "HEALTH" OR DE "ADVERSE childhood experiences" OR DE "CHILDREN'S

health" OR DE "ENVIRONMENTAL health" OR DE "EXERCISE" OR DE "FAMILY

health" OR DE "HEALTH & income" OR DE "HEALTH & race" OR DE "HEALTH

attitudes" OR DE "HEALTH of LGBTQ+ people" OR DE "HEALTH of older

people" OR DE "HEALTH of refugees" OR DE "HEALTH self-care" OR DE

"HEALTH status indicators" OR DE "MEN'S health" OR DE "MENTAL health" OR

DE "NUTRITION" OR DE "PHYSICAL fitness" OR DE "REPRODUCTIVE health" OR

DE "RURAL health" OR DE "SELF-neglect" OR DE "SEXUAL health" OR DE

"SLEEP" OR DE "TEENAGERS' health" OR DE "WOMEN'S health" Expanders -

Apply equivalent subjects

Search modes - Boolean/Phrase Interface - EBSCOhost Research Databases

Search Screen - Advanced Search

Database - SocINDEX with Full Text 70,101

S18 ((social* OR psychosoc*) N5 (determin* OR risk*) N7 ((employ* OR

unemploy* OR job OR jobs OR work OR worked OR works OR working) N5

(question* OR screen* OR tool* OR survey* OR ask* OR answer*)))

Expanders - Apply equivalent subjects

Search modes - Boolean/Phrase Interface - EBSCOhost Research Databases

Search Screen - Advanced Search

Database - SocINDEX with Full Text 34

S17 ((social* OR psychosoc*) N5 (determin* OR risk*) N7 (employ* OR

unemploy* OR job OR jobs OR work OR worked OR works OR working))

Expanders - Apply equivalent subjects

Search modes - Boolean/Phrase Interface - EBSCOhost Research Databases

Search Screen - Advanced Search

Database - SocINDEX with Full Text 1,293

S16 S2 AND S14 AND S15 Expanders - Apply equivalent subjects

Search modes - Boolean/Phrase Interface - EBSCOhost Research Databases

Search Screen - Advanced Search

Database - SocINDEX with Full Text 22

S15 (employ* OR unemploy* OR job OR jobs OR work OR worked OR works OR

working) Expanders - Apply equivalent subjects

Search modes - Boolean/Phrase Interface - EBSCOhost Research Databases

Search Screen - Advanced Search

Database - SocINDEX with Full Text 637,291

S14 S9 OR S12 OR S13 Expanders - Apply equivalent subjects

Search modes - Boolean/Phrase Interface - EBSCOhost Research Databases

Search Screen - Advanced Search

Database - SocINDEX with Full Text 4,445

S13 ((social* OR psychosoc*) N2 (determin* OR risk*) N5 (health* OR

diseas* or morbid* or mortal* or unhealth*)) Expanders - Apply

equivalent subjects

Search modes - Boolean/Phrase Interface - EBSCOhost Research Databases

Search Screen - Advanced Search

Database - SocINDEX with Full Text 1,757

S12 S6 OR S11 Expanders - Apply equivalent subjects

Search modes - Boolean/Phrase Interface - EBSCOhost Research Databases

Search Screen - Advanced Search

Database - SocINDEX with Full Text 1,031

S11 S9 AND S10 Expanders - Apply equivalent subjects

Search modes - Boolean/Phrase Interface - EBSCOhost Research Databases

Search Screen - Advanced Search

Database - SocINDEX with Full Text 92

S10 DE "HEALTH status indicators" OR DE "NUTRITIONAL status" OR DE

"HEALTH risk assessment" Expanders - Apply equivalent subjects

Search modes - Boolean/Phrase Interface - EBSCOhost Research Databases

Search Screen - Advanced Search

Database - SocINDEX with Full Text 7,315

S9 S7 OR S8 Expanders - Apply equivalent subjects

Search modes - Boolean/Phrase Interface - EBSCOhost Research Databases

Search Screen - Advanced Search

Database - SocINDEX with Full Text 2,075

S8 ((DE "ECONOMIC status" OR DE "CONSPICUOUS consumption" OR DE

"ECONOMIC conditions of older people")) AND (DE "SOCIAL status" OR DE

"ACHIEVED status" OR DE "ARCHITECTURE & social status" OR DE "ASCRIBED

status" OR DE "CREDENTIALISM" OR DE "FAME -- Social aspects" OR DE

"FASCISM & social status" OR DE "MARITAL status" OR DE "MASTER status"

OR DE "MENTAL health & social status" OR DE "RACE & social status" OR DE

"RELIGION & social status" OR DE "SOCIAL classes" OR DE "SOCIAL

conditions of students" OR DE "SOCIAL role" OR DE "SPEECH & social

status" OR DE "STATUS inconsistency" OR DE "STRUCTURAL social mobility"

OR DE "WIT & social status") Expanders - Apply equivalent subjects

Search modes - Boolean/Phrase Interface - EBSCOhost Research Databases

Search Screen - Advanced Search

Database - SocINDEX with Full Text 333

S7 DE "ECONOMIC status" OR DE "CONSPICUOUS consumption" OR DE "ECONOMIC

conditions of older people" OR DE "SOCIOECONOMIC status" Expanders -

Apply equivalent subjects

Search modes - Boolean/Phrase Interface - EBSCOhost Research Databases

Search Screen - Advanced Search

Database - SocINDEX with Full Text 2,075

S6 (DE "HEALTH & social status") OR (DE "HEALTH & economic status")

Expanders - Apply equivalent subjects

Search modes - Boolean/Phrase Interface - EBSCOhost Research Databases

Search Screen - Advanced Search

Database - SocINDEX with Full Text 947

S5 ((social* OR psychosoc*) N5 (determin* OR risk*) N7 (employment* OR

employed OR employee* OR unemploy* OR job OR jobs OR occupation*))

Expanders - Apply equivalent subjects

Search modes - Boolean/Phrase Interface - EBSCOhost Research Databases

Search Screen - Advanced Search

Database - SocINDEX with Full Text 385

S4 S2 AND S3 Expanders - Apply equivalent subjects

Search modes - Boolean/Phrase Interface - EBSCOhost Research Databases

Search Screen - Advanced Search

Database - SocINDEX with Full Text 21

S3 DE "EMPLOYMENT" OR DE "AGE & employment" OR DE "AGE discrimination

in employment" OR DE "CONTINGENT employment" OR DE "EMPLOYMENT

discrimination" OR DE "EMPLOYMENT of African Americans" OR DE

"EMPLOYMENT of Asian Americans" OR DE "EMPLOYMENT of Hispanic Americans"

OR DE "EMPLOYMENT of Native Americans" OR DE "EMPLOYMENT of black

people" OR DE "EMPLOYMENT of blind people" OR DE "EMPLOYMENT of college

graduates" OR DE "EMPLOYMENT of college students" OR DE "EMPLOYMENT of

ethnic groups" OR DE "EMPLOYMENT of ex-convicts" OR DE "EMPLOYMENT of

married people" OR DE "EMPLOYMENT of married women" OR DE "EMPLOYMENT of

men" OR DE "EMPLOYMENT of minorities" OR DE "EMPLOYMENT of mothers" OR

DE "EMPLOYMENT of older people" OR DE "EMPLOYMENT of older women" OR DE

"EMPLOYMENT of people with disabilities" OR DE "EMPLOYMENT of people

with mental disabilities" OR DE "EMPLOYMENT of poor people" OR DE

"EMPLOYMENT of pregnant women" OR DE "EMPLOYMENT of school dropouts" OR

DE "EMPLOYMENT of the mentally ill" OR DE "EMPLOYMENT of undocumented

immigrants" OR DE "EMPLOYMENT of veterans" OR DE "EMPLOYMENT of welfare

recipients" OR DE "EMPLOYMENT tenure" OR DE "FULL-time employment" OR DE

"JOB vacancies" OR DE "PART-time employment" OR DE "REVERSE

discrimination in employment" OR DE "SELF-employment" OR DE

"SUPPLEMENTARY employment" OR DE "TEMPORARY employment" OR DE

"UNEMPLOYMENT" OR DE "WOMEN'S employment" OR DE "YOUTH employment"

Expanders - Apply equivalent subjects

Search modes - Boolean/Phrase Interface - EBSCOhost Research Databases

Search Screen - Advanced Search

Database - SocINDEX with Full Text 39,670

S2 DE "HEALTH risk assessment" OR DE "MEDICAL screening" Expanders -

Apply equivalent subjects

Search modes - Boolean/Phrase Interface - EBSCOhost Research Databases

Search Screen - Advanced Search

Database - SocINDEX with Full Text 4,635

S1 ((screen* OR survey* OR question* OR ask*) N7 ((employ* OR unemploy*

OR job OR jobs OR work OR working OR occupation* OR career*) N3 (status*

OR situation* OR condition* OR histor*))) Expanders - Apply equivalent

subjects

Search modes - Boolean/Phrase Interface - EBSCOhost Research Databases

Search Screen - Advanced Search

Database - SocINDEX with Full Text 9,788

***************************

**[6]**

**Database: Ovid MEDLINE**

**Platform: Ovid**

**Date of Search: 4/14/21**

**# of Retrieved Results: 498**

**Search Strategy:**

1 ((screen* or survey* or question* or ask*) adj7 ((employ* or unemploy* or job or jobs or work or working or occupation* or career*) adj3 (status* or situation* or condition* or histor*))).mp. [mp=title, abstract, original title, name of substance word, subject heading word, floating sub-heading word, keyword heading word, organism supplementary concept word, protocol supplementary concept word, rare disease supplementary concept word, unique identifier, synonyms] (2867)

2 exp Mass Screening/ (132184)

3 exp Employment/ (89729)

4 2 and 3 (845)

5 AAFP Social Needs Screening Tool.mp. [mp=title, abstract, original title, name of substance word, subject heading word, floating sub-heading word, keyword heading word, organism supplementary concept word, protocol supplementary concept word, rare disease supplementary concept word, unique identifier, synonyms] (0)

6 Social Needs Screening Tool*.mp. [mp=title, abstract, original title, name of substance word, subject heading word, floating sub-heading word, keyword heading word, organism supplementary concept word, protocol supplementary concept word, rare disease supplementary concept word, unique identifier, synonyms] (3)

7 AccessHealth Spartanburg Screening Tool.mp. [mp=title, abstract, original title, name of substance word, subject heading word, floating sub-heading word, keyword heading word, organism supplementary concept word, protocol supplementary concept word, rare disease supplementary concept word, unique identifier, synonyms] (0)

8 ((AccessHealth or Spartanburg) adj3 Screening Tool*).mp. [mp=title, abstract, original title, name of substance word, subject heading word, floating sub-heading word, keyword heading word, organism supplementary concept word, protocol supplementary concept word, rare disease supplementary concept word, unique identifier, synonyms] (0)

9 Accountable Health Communities Health-Related Social Needs Screening Tool.mp. [mp=title, abstract, original title, name of substance word, subject heading word, floating sub-heading word, keyword heading word, organism supplementary concept word, protocol supplementary concept word, rare disease supplementary concept word, unique identifier, synonyms] (0)

10 Boston Medical Center-Thrive Screening Tool.mp. [mp=title, abstract, original title, name of substance word, subject heading word, floating sub-heading word, keyword heading word, organism supplementary concept word, protocol supplementary concept word, rare disease supplementary concept word, unique identifier, synonyms] (0)

11 HealthBegins Upstream Risk Screening Tool.mp. [mp=title, abstract, original title, name of substance word, subject heading word, floating sub-heading word, keyword heading word, organism supplementary concept word, protocol supplementary concept word, rare disease supplementary concept word, unique identifier, synonyms] (0)

12 Medical-Legal Partnership IHELLP.mp. [mp=title, abstract, original title, name of substance word, subject heading word, floating sub-heading word, keyword heading word, organism supplementary concept word, protocol supplementary concept word, rare disease supplementary concept word, unique identifier, synonyms] (0)

13 PRAPARE.mp. [mp=title, abstract, original title, name of substance word, subject heading word, floating sub-heading word, keyword heading word, organism supplementary concept word, protocol supplementary concept word, rare disease supplementary concept word, unique identifier, synonyms] (5)

14 WellRx Toolkit.mp. [mp=title, abstract, original title, name of substance word, subject heading word, floating sub-heading word, keyword heading word, organism supplementary concept word, protocol supplementary concept word, rare disease supplementary concept word, unique identifier, synonyms] (0)

15 We Care Screening Tool.mp. [mp=title, abstract, original title, name of substance word, subject heading word, floating sub-heading word, keyword heading word, organism supplementary concept word, protocol supplementary concept word, rare disease supplementary concept word, unique identifier, synonyms] (1)

16 iscreen.mp. [mp=title, abstract, original title, name of substance word, subject heading word, floating sub-heading word, keyword heading word, organism supplementary concept word, protocol supplementary concept word, rare disease supplementary concept word, unique identifier, synonyms] (14)

17 Family fIRST.mp. [mp=title, abstract, original title, name of substance word, subject heading word, floating sub-heading word, keyword heading word, organism supplementary concept word, protocol supplementary concept word, rare disease supplementary concept word, unique identifier, synonyms] (185)

18 screen*.mp. [mp=title, abstract, original title, name of substance word, subject heading word, floating sub-heading word, keyword heading word, organism supplementary concept word, protocol supplementary concept word, rare disease supplementary concept word, unique identifier, synonyms] (878769)

19 17 and 18 (9)

20 ((social* or psychosoc*) adj5 (determin* or risk*) adj7 (employment* or employed or employee* or unemploy* or job or jobs or occupation*)).mp. [mp=title, abstract, original title, name of substance word, subject heading word, floating sub-heading word, keyword heading word, organism supplementary concept word, protocol supplementary concept word, rare disease supplementary concept word, unique identifier, synonyms] (859)

21 exp "Social Determinants of Health"/ (4037)

22 ((social* or psychosoc*) adj2 (determin* or risk*) adj5 (health* or unhealth*)).mp. [mp=title, abstract, original title, name of substance word, subject heading word, floating sub-heading word, keyword heading word, organism supplementary concept word, protocol supplementary concept word, rare disease supplementary concept word, unique identifier, synonyms] (10533)

23 21 or 22 (10533)

24 exp Socioeconomic Factors/ (464194)

25 (employ* or unemploy* or job or jobs or work or worked or works or working).mp. [mp=title, abstract, original title, name of substance word, subject heading word, floating sub-heading word, keyword heading word, organism supplementary concept word, protocol supplementary concept word, rare disease supplementary concept word, unique identifier, synonyms] (2011283)

26 23 or 24 (471288)

27 2 and 25 and 26 (1341)

28 ((social* or psychosoc*) adj5 (determin* or risk*) adj7 (employ* or unemploy* or job or jobs or work or worked or works or working)).mp. (1497)

29 ((social* or psychosoc*) adj5 (determin* or risk*) adj7 ((employ* or unemploy* or job or jobs or work or worked or works or working) adj5 (question* or screen* or tool* or survey* or ask* or answer*))).mp. (49)

30 exp Data Collection/ (2273485)

31 exp Health Status/ (358414)

32 26 or 31 (780878)

33 30 and 32 (316860)

34 3 and 33 (26364)

35 2 and 34 (560)

36 (question* or screen* or tool* or survey* or ask* or answer*).mp. [mp=title, abstract, original title, name of substance word, subject heading word, floating sub-heading word, keyword heading word, organism supplementary concept word, protocol supplementary concept word, rare disease supplementary concept word, unique identifier, synonyms] (3233026)

37 28 and 36 (781)

38 1 or 4 or 29 or 35 or 37 (4452)

39 exp pregnancy/ (913911)

40 exp pregnancy complications/ (436209)

41 exp pregnant women/ (9239)

42 exp maternal health services/ (51699)

43 exp maternal exposure/ (9828)

44 exp maternal health/ (1698)

45 39 or 40 or 41 or 42 or 43 or 44 (954861)

46 (pregnan* or obstet* or childbear* or perinatal* or peri-natal* or prenatal* or pre-natal* or (family adj2 planning) or ((maternal* or mother*) adj3 (health* or welfar* or expos* or risk*))).mp. [mp=title, abstract, original title, name of substance word, subject heading word, floating sub-heading word, keyword heading word, organism supplementary concept word, protocol supplementary concept word, rare disease supplementary concept word, unique identifier, synonyms] (1184818)

47 45 or 46 (1222003)

48 38 and 47 (180)

49 5 or 6 or 7 or 8 or 9 or 10 or 11 or 12 or 13 or 14 or 15 or 16 or 17 (208)

50 47 and 49 (10)

51 48 or 50 (190)

52 20 or 23 (11192)

53 2 or 36 (3240818)

54 47 and 52 and 53 (318)

55 54 not 51 (308)

56 4 and 33 (560)

57 3 and 23 and 30 (153)

58 56 or 57 (712)

59 58 not (51 or 54) (675)

60 exp "Delivery of Health Care"/ (1117496)

61 59 and 60 (149)

62 exp risk/ (1257864)

63 59 and 62 (169)

64 61 or 63 (279)

65 51 or 54 (498)

***************************

**[7]**

**Database: APA PsycInfo**

**Platform: Ovid**

**Date of Search: 4/14/21**

**# of Retrieved Results: 391**

**Search Strategy:**

1 ((screen* or survey* or question* or ask*) adj7 ((employ* or unemploy* or job or jobs or work or working or occupation* or career*) adj3 (status* or situation* or condition* or histor*))).mp. [mp=title, abstract, heading word, table of contents, key concepts, original title, tests & measures, mesh] (1784)

2 exp Screening/ (33387)

3 exp Employment/ (27965)

4 2 and 3 (125)

5 AAFP Social Needs Screening Tool.mp. [mp=title, abstract, heading word, table of contents, key concepts, original title, tests & measures, mesh] (0)

6 Social Needs Screening Tool*.mp. [mp=title, abstract, heading word, table of contents, key concepts, original title, tests & measures, mesh] (0)

7 AccessHealth Spartanburg Screening Tool.mp. [mp=title, abstract, heading word, table of contents, key concepts, original title, tests & measures, mesh] (0)

8 ((AccessHealth or Spartanburg) adj3 Screening Tool*).mp. [mp=title, abstract, heading word, table of contents, key concepts, original title, tests & measures, mesh] (0)

9 Accountable Health Communities Health-Related Social Needs Screening Tool.mp. [mp=title, abstract, heading word, table of contents, key concepts, original title, tests & measures, mesh] (0)

10 Boston Medical Center-Thrive Screening Tool.mp. [mp=title, abstract, heading word, table of contents, key concepts, original title, tests & measures, mesh] (0)

11 HealthBegins Upstream Risk Screening Tool.mp. [mp=title, abstract, heading word, table of contents, key concepts, original title, tests & measures, mesh] (0)

12 Medical-Legal Partnership IHELLP.mp. [mp=title, abstract, heading word, table of contents, key concepts, original title, tests & measures, mesh] (0)

13 PRAPARE.mp. [mp=title, abstract, heading word, table of contents, key concepts, original title, tests & measures, mesh] (2)

14 WellRx Toolkit.mp. [mp=title, abstract, heading word, table of contents, key concepts, original title, tests & measures, mesh] (0)

15 We Care Screening Tool.mp. [mp=title, abstract, heading word, table of contents, key concepts, original title, tests & measures, mesh] (2)

16 iscreen.mp. [mp=title, abstract, heading word, table of contents, key concepts, original title, tests & measures, mesh] (1)

17 Family fIRST.mp. [mp=title, abstract, heading word, table of contents, key concepts, original title, tests & measures, mesh] (106)

18 screen*.mp. [mp=title, abstract, heading word, table of contents, key concepts, original title, tests & measures, mesh] (124317)

19 17 and 18 (2)

20 ((social* or psychosoc*) adj5 (determin* or risk*) adj7 (employment* or employed or employee* or unemploy* or job or jobs or occupation*)).mp. [mp=title, abstract, heading word, table of contents, key concepts, original title, tests & measures, mesh] (1338)

21 exp Socioeconomic Factors/ (86241)

22 exp sociocultural factors/ (122673)

23 21 or 22 (203268)

24 exp health disparities/ or exp health/ (351774)

25 23 and 24 (24615)

26 ((social* or psychosoc*) adj2 (determin* or risk*) adj5 (health* or unhealth*)).mp. [mp=title, abstract, heading word, table of contents, key concepts, original title, tests & measures, mesh] (6641)

27 25 or 26 (30585)

28 exp Socioeconomic Factors/ (86241)

29 exp sociocultural factors/ (122673)

30 (employ* or unemploy* or job or jobs or work or worked or works or working).mp. [mp=title, abstract, heading word, table of contents, key concepts, original title, tests & measures, mesh] (936416)

31 27 or 28 (102494)

32 2 and 30 and 31 (231)

33 ((social* or psychosoc*) adj5 (determin* or risk*) adj7 (employ* or unemploy* or job or jobs or work or worked or works or working)).mp. (1857)

34 ((social* or psychosoc*) adj5 (determin* or risk*) adj7 ((employ* or unemploy* or job or jobs or work or worked or works or working) adj5 (question* or screen* or tool* or survey* or ask* or answer*))).mp. (125)

35 exp Data Collection/ (70408)

36 exp Health Status/ (47386)

37 31 or 36 (147384)

38 35 and 37 (1801)

39 3 and 38 (318)

40 2 and 39 (4)

41 (question* or screen* or tool* or survey* or ask* or answer*).mp. [mp=title, abstract, heading word, table of contents, key concepts, original title, tests & measures, mesh] (1296877)

42 33 and 41 (844)

43 1 or 4 or 34 or 40 or 42 (2715)

44 exp pregnancy/ (43548)

45 exp prenatal development/ (7818)

46 exp obstetrics/ (2921)

47 exp perinatal period/ (3098)

48 44 or 45 or 46 or 47 (52755)

49 (pregnan* or obstet* or childbear* or perinatal* or peri-natal* or prenatal* or pre-natal* or (family adj2 planning) or ((maternal* or mother*) adj3 (health* or welfar* or expos* or risk* or harm* or advers* or affect* or effect* or impact* or inhibit* or interrupt* or interfer*))).mp. [mp=title, abstract, heading word, table of contents, key concepts, original title, tests & measures, mesh] (108513)

50 48 or 49 (119765)

51 43 and 50 (78)

52 5 or 6 or 7 or 8 or 9 or 10 or 11 or 12 or 13 or 14 or 15 or 16 or 17 (111)

53 50 and 52 (9)

54 51 or 53 (87)

55 20 or 27 (31549)

56 2 or 41 (1300114)

57 50 and 55 and 56 (883)

58 57 not 54 (850)

59 48 and 55 and 56 (316)

60 54 or 59 (391)

***************************

**[8]**

**Database: SocINDEX**

**Platform: EBSCO**

**Date of Search: 2/16/22**

**# of Retrieved Results: 10**

**Search Strategy:**

Search ID# Search Terms Search Options Last Run Via Results

S36 S34 AND S35 Expanders - Apply equivalent subjects

Search modes - Boolean/Phrase Interface - EBSCOhost Research Databases

Search Screen - Advanced Search

Database - SocINDEX with Full Text 10

S35 ZD(202105* or 202106 or 202107* or 202108* or 202109* or 20211* or

2022*) Expanders - Apply equivalent subjects

Search modes - Boolean/Phrase Interface - EBSCOhost Research Databases

Search Screen - Advanced Search

Database - SocINDEX with Full Text 43,753

S34 S27 AND S33 Expanders - Apply equivalent subjects

Search modes - Boolean/Phrase Interface - EBSCOhost Research Databases

Search Screen - Advanced Search

Database - SocINDEX with Full Text 350

S33 S31 OR S32 Expanders - Apply equivalent subjects

Search modes - Boolean/Phrase Interface - EBSCOhost Research Databases

Search Screen - Advanced Search

Database - SocINDEX with Full Text 45,658

S32 (pregnan* OR obstet* OR childbear* OR perinatal* OR peri-natal* OR

prenatal* OR pre-natal* OR (family N2 planning) OR ((maternal* OR

mother*) N3 (health* OR welfar* OR expos* OR risk*))) Expanders - Apply

equivalent subjects

Search modes - Boolean/Phrase Interface - EBSCOhost Research Databases

Search Screen - Advanced Search

Database - SocINDEX with Full Text 44,459

S31 S28 OR S29 OR S30 Expanders - Apply equivalent subjects

Search modes - Boolean/Phrase Interface - EBSCOhost Research Databases

Search Screen - Advanced Search

Database - SocINDEX with Full Text 12,599

S30 DE "OBSTETRICS" OR DE "CHILDBIRTH" OR DE "MIDWIFERY" Expanders -

Apply equivalent subjects

Search modes - Boolean/Phrase Interface - EBSCOhost Research Databases

Search Screen - Advanced Search

Database - SocINDEX with Full Text 4,329

S29 DE "MATERNAL health" Expanders - Apply equivalent subjects

Search modes - Boolean/Phrase Interface - EBSCOhost Research Databases

Search Screen - Advanced Search

Database - SocINDEX with Full Text 525

S28 DE "PREGNANCY" OR DE "EXTRAMARITAL pregnancy" OR DE "MALE

pregnancy" OR DE "MULTIPLE pregnancy" OR DE "PARITY (Obstetrics)" OR DE

"PREGNANT women" OR DE "TEENAGE pregnancy" OR DE "UNPLANNED pregnancy"

OR DE "UNWANTED pregnancy" Expanders - Apply equivalent subjects

Search modes - Boolean/Phrase Interface - EBSCOhost Research Databases

Search Screen - Advanced Search

Database - SocINDEX with Full Text 8,903

S27 S1 OR S4 OR S24 OR S26 Expanders - Apply equivalent subjects

Search modes - Boolean/Phrase Interface - EBSCOhost Research Databases

Search Screen - Advanced Search

Database - SocINDEX with Full Text 10,464

S26 S17 AND S25 Expanders - Apply equivalent subjects

Search modes - Boolean/Phrase Interface - EBSCOhost Research Databases

Search Screen - Advanced Search

Database - SocINDEX with Full Text 397

S25 (question* OR screen* OR tool* OR survey* OR ask* OR answer*)

Expanders - Apply equivalent subjects

Search modes - Boolean/Phrase Interface - EBSCOhost Research Databases

Search Screen - Advanced Search

Database - SocINDEX with Full Text 421,268

S24 S16 OR S18 OR S23 Expanders - Apply equivalent subjects

Search modes - Boolean/Phrase Interface - EBSCOhost Research Databases

Search Screen - Advanced Search

Database - SocINDEX with Full Text 79

S23 S3 AND S22 Expanders - Apply equivalent subjects

Search modes - Boolean/Phrase Interface - EBSCOhost Research Databases

Search Screen - Advanced Search

Database - SocINDEX with Full Text 22

S22 S20 AND S21 Expanders - Apply equivalent subjects

Search modes - Boolean/Phrase Interface - EBSCOhost Research Databases

Search Screen - Advanced Search

Database - SocINDEX with Full Text 836

S21 DE "SOCIAL science research" OR DE "ACTION research" OR DE

"COMMUNITY life research" OR DE "COMMUNITY-based participatory research"

OR DE "ETHNOSTATISTICS" OR DE "EVALUATION research (Social action

programs)" OR DE "ORGANIZATIONAL research" OR DE "PSYCHOLOGICAL

research" OR DE "SOCIAL network analysis" OR DE "SOCIAL surveys" OR DE

"SOCIOLOGICAL imagination" OR DE "SOCIOLOGICAL research" OR DE

"STATISTICS on social sciences" OR DE "TELEPHONE surveys" Expanders -

Apply equivalent subjects

Search modes - Boolean/Phrase Interface - EBSCOhost Research Databases

Search Screen - Advanced Search

Database - SocINDEX with Full Text 28,600

S20 S14 OR S19 Expanders - Apply equivalent subjects

Search modes - Boolean/Phrase Interface - EBSCOhost Research Databases

Search Screen - Advanced Search

Database - SocINDEX with Full Text 76,209

S19 DE "HEALTH" OR DE "ADVERSE childhood experiences" OR DE "CHILDREN'S

health" OR DE "ENVIRONMENTAL health" OR DE "EXERCISE" OR DE "FAMILY

health" OR DE "HEALTH & income" OR DE "HEALTH & race" OR DE "HEALTH

attitudes" OR DE "HEALTH of LGBTQ+ people" OR DE "HEALTH of older

people" OR DE "HEALTH of refugees" OR DE "HEALTH self-care" OR DE

"HEALTH status indicators" OR DE "MEN'S health" OR DE "MENTAL health" OR

DE "NUTRITION" OR DE "PHYSICAL fitness" OR DE "REPRODUCTIVE health" OR

DE "RURAL health" OR DE "SELF-neglect" OR DE "SEXUAL health" OR DE

"SLEEP" OR DE "TEENAGERS' health" OR DE "WOMEN'S health" Expanders -

Apply equivalent subjects

Search modes - Boolean/Phrase Interface - EBSCOhost Research Databases

Search Screen - Advanced Search

Database - SocINDEX with Full Text 72,656

S18 ((social* OR psychosoc*) N5 (determin* OR risk*) N7 ((employ* OR

unemploy* OR job OR jobs OR work OR worked OR works OR working) N5

(question* OR screen* OR tool* OR survey* OR ask* OR answer*)))

Expanders - Apply equivalent subjects

Search modes - Boolean/Phrase Interface - EBSCOhost Research Databases

Search Screen - Advanced Search

Database - SocINDEX with Full Text 34

S17 ((social* OR psychosoc*) N5 (determin* OR risk*) N7 (employ* OR

unemploy* OR job OR jobs OR work OR worked OR works OR working))

Expanders - Apply equivalent subjects

Search modes - Boolean/Phrase Interface - EBSCOhost Research Databases

Search Screen - Advanced Search

Database - SocINDEX with Full Text 1,352

S16 S2 AND S14 AND S15 Expanders - Apply equivalent subjects

Search modes - Boolean/Phrase Interface - EBSCOhost Research Databases

Search Screen - Advanced Search

Database - SocINDEX with Full Text 23

S15 (employ* OR unemploy* OR job OR jobs OR work OR worked OR works OR

working) Expanders - Apply equivalent subjects

Search modes - Boolean/Phrase Interface - EBSCOhost Research Databases

Search Screen - Advanced Search

Database - SocINDEX with Full Text 651,872

S14 S9 OR S12 OR S13 Expanders - Apply equivalent subjects

Search modes - Boolean/Phrase Interface - EBSCOhost Research Databases

Search Screen - Advanced Search

Database - SocINDEX with Full Text 4,842

S13 ((social* OR psychosoc*) N2 (determin* OR risk*) N5 (health* OR

diseas* or morbid* or mortal* or unhealth*)) Expanders - Apply

equivalent subjects

Search modes - Boolean/Phrase Interface - EBSCOhost Research Databases

Search Screen - Advanced Search

Database - SocINDEX with Full Text 1,975

S12 S6 OR S11 Expanders - Apply equivalent subjects

Search modes - Boolean/Phrase Interface - EBSCOhost Research Databases

Search Screen - Advanced Search

Database - SocINDEX with Full Text 1,042

S11 S9 AND S10 Expanders - Apply equivalent subjects

Search modes - Boolean/Phrase Interface - EBSCOhost Research Databases

Search Screen - Advanced Search

Database - SocINDEX with Full Text 106

S10 DE "HEALTH status indicators" OR DE "NUTRITIONAL status" OR DE

"HEALTH risk assessment" Expanders - Apply equivalent subjects

Search modes - Boolean/Phrase Interface - EBSCOhost Research Databases

Search Screen - Advanced Search

Database - SocINDEX with Full Text 7,814

S9 S7 OR S8 Expanders - Apply equivalent subjects

Search modes - Boolean/Phrase Interface - EBSCOhost Research Databases

Search Screen - Advanced Search

Database - SocINDEX with Full Text 2,261

S8 ((DE "ECONOMIC status" OR DE "CONSPICUOUS consumption" OR DE

"ECONOMIC conditions of older people")) AND (DE "SOCIAL status" OR DE

"ACHIEVED status" OR DE "ARCHITECTURE & social status" OR DE "ASCRIBED

status" OR DE "CREDENTIALISM" OR DE "FAME -- Social aspects" OR DE

"FASCISM & social status" OR DE "MARITAL status" OR DE "MASTER status"

OR DE "MENTAL health & social status" OR DE "RACE & social status" OR DE

"RELIGION & social status" OR DE "SOCIAL classes" OR DE "SOCIAL

conditions of students" OR DE "SOCIAL role" OR DE "SPEECH & social

status" OR DE "STATUS inconsistency" OR DE "STRUCTURAL social mobility"

OR DE "WIT & social status") Expanders - Apply equivalent subjects

Search modes - Boolean/Phrase Interface - EBSCOhost Research Databases

Search Screen - Advanced Search

Database - SocINDEX with Full Text 346

S7 DE "ECONOMIC status" OR DE "CONSPICUOUS consumption" OR DE "ECONOMIC

conditions of older people" OR DE "SOCIOECONOMIC status" Expanders -

Apply equivalent subjects

Search modes - Boolean/Phrase Interface - EBSCOhost Research Databases

Search Screen - Advanced Search

Database - SocINDEX with Full Text 2,261

S6 (DE "HEALTH & social status") OR (DE "HEALTH & economic status")

Expanders - Apply equivalent subjects

Search modes - Boolean/Phrase Interface - EBSCOhost Research Databases

Search Screen - Advanced Search

Database - SocINDEX with Full Text 944

S5 ((social* OR psychosoc*) N5 (determin* OR risk*) N7 (employment* OR

employed OR employee* OR unemploy* OR job OR jobs OR occupation*))

Expanders - Apply equivalent subjects

Search modes - Boolean/Phrase Interface - EBSCOhost Research Databases

Search Screen - Advanced Search

Database - SocINDEX with Full Text 403

S4 S2 AND S3 Expanders - Apply equivalent subjects

Search modes - Boolean/Phrase Interface - EBSCOhost Research Databases

Search Screen - Advanced Search

Database - SocINDEX with Full Text 21

S3 DE "EMPLOYMENT" OR DE "AGE & employment" OR DE "AGE discrimination

in employment" OR DE "CONTINGENT employment" OR DE "EMPLOYMENT

discrimination" OR DE "EMPLOYMENT of African Americans" OR DE

"EMPLOYMENT of Asian Americans" OR DE "EMPLOYMENT of Hispanic Americans"

OR DE "EMPLOYMENT of Native Americans" OR DE "EMPLOYMENT of black

people" OR DE "EMPLOYMENT of blind people" OR DE "EMPLOYMENT of college

graduates" OR DE "EMPLOYMENT of college students" OR DE "EMPLOYMENT of

ethnic groups" OR DE "EMPLOYMENT of ex-convicts" OR DE "EMPLOYMENT of

married people" OR DE "EMPLOYMENT of married women" OR DE "EMPLOYMENT of

men" OR DE "EMPLOYMENT of minorities" OR DE "EMPLOYMENT of mothers" OR

DE "EMPLOYMENT of older people" OR DE "EMPLOYMENT of older women" OR DE

"EMPLOYMENT of people with disabilities" OR DE "EMPLOYMENT of people

with mental disabilities" OR DE "EMPLOYMENT of poor people" OR DE

"EMPLOYMENT of pregnant women" OR DE "EMPLOYMENT of school dropouts" OR

DE "EMPLOYMENT of the mentally ill" OR DE "EMPLOYMENT of undocumented

immigrants" OR DE "EMPLOYMENT of veterans" OR DE "EMPLOYMENT of welfare

recipients" OR DE "EMPLOYMENT tenure" OR DE "FULL-time employment" OR DE

"JOB vacancies" OR DE "PART-time employment" OR DE "REVERSE

discrimination in employment" OR DE "SELF-employment" OR DE

"SUPPLEMENTARY employment" OR DE "TEMPORARY employment" OR DE

"UNEMPLOYMENT" OR DE "WOMEN'S employment" OR DE "YOUTH employment"

Expanders - Apply equivalent subjects

Search modes - Boolean/Phrase Interface - EBSCOhost Research Databases

Search Screen - Advanced Search

Database - SocINDEX with Full Text 40,511

S2 DE "HEALTH risk assessment" OR DE "MEDICAL screening" Expanders -

Apply equivalent subjects

Search modes - Boolean/Phrase Interface - EBSCOhost Research Databases

Search Screen - Advanced Search

Database - SocINDEX with Full Text 4,743

S1 ((screen* OR survey* OR question* OR ask*) N7 ((employ* OR unemploy*

OR job OR jobs OR work OR working OR occupation* OR career*) N3 (status*

OR situation* OR condition* OR histor*))) Expanders - Apply equivalent

subjects

Search modes - Boolean/Phrase Interface - EBSCOhost Research Databases

Search Screen - Advanced Search

Database - SocINDEX with Full Text 10,066

***************************

**[9]**

**Database: Embase**

**Platform: Embase.com**

**Date of Search: 2/15/22**

**# of Retrieved Results: 19**

**Search Strategy:**

((('mass screening'/exp AND (((screen* OR survey* OR question* OR ask*) NEAR/7 (employ* OR unemploy* OR job OR jobs OR work OR working OR occupation* OR career*) OR ('mass screening'/exp AND 'employment'/exp) OR ((social* OR psychosoc*) NEAR/5 (determin* OR risk*) AND (determin* OR risk*) NEAR/7 (employ* OR unemploy* OR job OR jobs OR work OR worked OR works OR working) AND (employ* OR unemploy* OR job OR jobs OR work OR worked OR works OR working) NEAR/5 (question* OR screen* OR tool* OR survey* OR ask* OR answer*)) OR ('mass screening'/exp AND ('employment'/exp AND ('data collection method'/exp AND ((('social determinants of health'/exp OR ((social* OR psychosoc*) NEAR/2 (determin* OR risk*) AND (determin* OR risk*) NEAR/5 (health* OR unhealth*))) OR 'socioeconomics'/exp) OR ('health status'/exp OR 'health status indicator'/exp))))) OR (((social* OR psychosoc*) NEAR/5 (determin* OR risk*) AND (determin* OR risk*) NEAR/7 (employ* OR unemploy* OR job OR jobs OR work OR worked OR works OR working)) AND (question*:ti,ab,de,tn OR screen*:ti,ab,de,tn OR tool*:ti,ab,de,tn OR survey*:ti,ab,de,tn OR ask*:ti,ab,de,tn OR answer*:ti,ab,de,tn))) AND (('pregnancy'/exp OR 'pregnancy complication'/exp OR 'maternal care'/exp OR 'maternal exposure'/exp OR 'obstetrics'/exp OR 'obstetric procedure'/exp) OR (pregnant* OR obstetric* OR maternal* OR perinat* OR prenat*)))) AND ('socioeconomics'/exp AND 'data collection method'/exp AND (((screen* OR survey* OR question* OR ask*) NEAR/7 (employ* OR unemploy* OR job OR jobs OR work OR working OR occupation* OR career*) OR ('mass screening'/exp AND 'employment'/exp) OR ((social* OR psychosoc*) NEAR/5 (determin* OR risk*) AND (determin* OR risk*) NEAR/7 (employ* OR unemploy* OR job OR jobs OR work OR worked OR works OR working) AND (employ* OR unemploy* OR job OR jobs OR work OR worked OR works OR working) NEAR/5 (question* OR screen* OR tool* OR survey* OR ask* OR answer*)) OR ('mass screening'/exp AND ('employment'/exp AND ('data collection method'/exp AND ((('social determinants of health'/exp OR ((social* OR psychosoc*) NEAR/2 (determin* OR risk*) AND (determin* OR risk*) NEAR/5 (health* OR unhealth*))) OR 'socioeconomics'/exp) OR ('health status'/exp OR 'health status indicator'/exp))))) OR (((social* OR psychosoc*) NEAR/5 (determin* OR risk*) AND (determin* OR risk*) NEAR/7 (employ* OR unemploy* OR job OR jobs OR work OR worked OR works OR working)) AND (question*:ti,ab,de,tn OR screen*:ti,ab,de,tn OR tool*:ti,ab,de,tn OR survey*:ti,ab,de,tn OR ask*:ti,ab,de,tn OR answer*:ti,ab,de,tn))) AND (('pregnancy'/exp OR 'pregnancy complication'/exp OR 'maternal care'/exp OR 'maternal exposure'/exp OR 'obstetrics'/exp OR 'obstetric procedure'/exp) OR (pregnant* OR obstetric* OR maternal* OR perinat* OR prenat*))))) OR (('mass screening'/exp AND (((screen* OR survey* OR question* OR ask*) NEAR/7 (employ* OR unemploy* OR job OR jobs OR work OR working OR occupation* OR career*) OR ('mass screening'/exp AND 'employment'/exp) OR ((social* OR psychosoc*) NEAR/5 (determin* OR risk*) AND (determin* OR risk*) NEAR/7 (employ* OR unemploy* OR job OR jobs OR work OR worked OR works OR working) AND (employ* OR unemploy* OR job OR jobs OR work OR worked OR works OR working) NEAR/5 (question* OR screen* OR tool* OR survey* OR ask* OR answer*)) OR ('mass screening'/exp AND ('employment'/exp AND ('data collection method'/exp AND ((('social determinants of health'/exp OR ((social* OR psychosoc*) NEAR/2 (determin* OR risk*) AND (determin* OR risk*) NEAR/5 (health* OR unhealth*))) OR 'socioeconomics'/exp) OR ('health status'/exp OR 'health status indicator'/exp))))) OR (((social* OR psychosoc*) NEAR/5 (determin* OR risk*) AND (determin* OR risk*) NEAR/7 (employ* OR unemploy* OR job OR jobs OR work OR worked OR works OR working)) AND (question*:ti,ab,de,tn OR screen*:ti,ab,de,tn OR tool*:ti,ab,de,tn OR survey*:ti,ab,de,tn OR ask*:ti,ab,de,tn OR answer*:ti,ab,de,tn))) AND (('pregnancy'/exp OR 'pregnancy complication'/exp OR 'maternal care'/exp OR 'maternal exposure'/exp OR 'obstetrics'/exp OR 'obstetric procedure'/exp) OR (pregnant* OR obstetric* OR maternal* OR perinat* OR prenat*)))) AND ('employment'/exp AND (((screen* OR survey* OR question* OR ask*) NEAR/7 (employ* OR unemploy* OR job OR jobs OR work OR working OR occupation* OR career*) OR ('mass screening'/exp AND 'employment'/exp) OR ((social* OR psychosoc*) NEAR/5 (determin* OR risk*) AND (determin* OR risk*) NEAR/7 (employ* OR unemploy* OR job OR jobs OR work OR worked OR works OR working) AND (employ* OR unemploy* OR job OR jobs OR work OR worked OR works OR working) NEAR/5 (question* OR screen* OR tool* OR survey* OR ask* OR answer*)) OR ('mass screening'/exp AND ('employment'/exp AND ('data collection method'/exp AND ((('social determinants of health'/exp OR ((social* OR psychosoc*) NEAR/2 (determin* OR risk*) AND (determin* OR risk*) NEAR/5 (health* OR unhealth*))) OR 'socioeconomics'/exp) OR ('health status'/exp OR 'health status indicator'/exp))))) OR (((social* OR psychosoc*) NEAR/5 (determin* OR risk*) AND (determin* OR risk*) NEAR/7 (employ* OR unemploy* OR job OR jobs OR work OR worked OR works OR working)) AND (question*:ti,ab,de,tn OR screen*:ti,ab,de,tn OR tool*:ti,ab,de,tn OR survey*:ti,ab,de,tn OR ask*:ti,ab,de,tn OR answer*:ti,ab,de,tn))) AND (('pregnancy'/exp OR 'pregnancy complication'/exp OR 'maternal care'/exp OR 'maternal exposure'/exp OR 'obstetrics'/exp OR 'obstetric procedure'/exp) OR (pregnant* OR obstetric* OR maternal* OR perinat* OR prenat*))))) OR (('socioeconomics'/exp AND 'data collection method'/exp AND (((screen* OR survey* OR question* OR ask*) NEAR/7 (employ* OR unemploy* OR job OR jobs OR work OR working OR occupation* OR career*) OR ('mass screening'/exp AND 'employment'/exp) OR ((social* OR psychosoc*) NEAR/5 (determin* OR risk*) AND (determin* OR risk*) NEAR/7 (employ* OR unemploy* OR job OR jobs OR work OR worked OR works OR working) AND (employ* OR unemploy* OR job OR jobs OR work OR worked OR works OR working) NEAR/5 (question* OR screen* OR tool* OR survey* OR ask* OR answer*)) OR ('mass screening'/exp AND ('employment'/exp AND ('data collection method'/exp AND ((('social determinants of health'/exp OR ((social* OR psychosoc*) NEAR/2 (determin* OR risk*) AND (determin* OR risk*) NEAR/5 (health* OR unhealth*))) OR 'socioeconomics'/exp) OR ('health status'/exp OR 'health status indicator'/exp))))) OR (((social* OR psychosoc*) NEAR/5 (determin* OR risk*) AND (determin* OR risk*) NEAR/7 (employ* OR unemploy* OR job OR jobs OR work OR worked OR works OR working)) AND (question*:ti,ab,de,tn OR screen*:ti,ab,de,tn OR tool*:ti,ab,de,tn OR survey*:ti,ab,de,tn OR ask*:ti,ab,de,tn OR answer*:ti,ab,de,tn))) AND (('pregnancy'/exp OR 'pregnancy complication'/exp OR 'maternal care'/exp OR 'maternal exposure'/exp OR 'obstetrics'/exp OR 'obstetric procedure'/exp) OR (pregnant* OR obstetric* OR maternal* OR perinat* OR prenat*)))) AND ('employment'/exp AND (((screen* OR survey* OR question* OR ask*) NEAR/7 (employ* OR unemploy* OR job OR jobs OR work OR working OR occupation* OR career*) OR ('mass screening'/exp AND 'employment'/exp) OR ((social* OR psychosoc*) NEAR/5 (determin* OR risk*) AND (determin* OR risk*) NEAR/7 (employ* OR unemploy* OR job OR jobs OR work OR worked OR works OR working) AND (employ* OR unemploy* OR job OR jobs OR work OR worked OR works OR working) NEAR/5 (question* OR screen* OR tool* OR survey* OR ask* OR answer*)) OR ('mass screening'/exp AND ('employment'/exp AND ('data collection method'/exp AND ((('social determinants of health'/exp OR ((social* OR psychosoc*) NEAR/2 (determin* OR risk*) AND (determin* OR risk*) NEAR/5 (health* OR unhealth*))) OR 'socioeconomics'/exp) OR ('health status'/exp OR 'health status indicator'/exp))))) OR (((social* OR psychosoc*) NEAR/5 (determin* OR risk*) AND (determin* OR risk*) NEAR/7 (employ* OR unemploy* OR job OR jobs OR work OR worked OR works OR working)) AND (question*:ti,ab,de,tn OR screen*:ti,ab,de,tn OR tool*:ti,ab,de,tn OR survey*:ti,ab,de,tn OR ask*:ti,ab,de,tn OR answer*:ti,ab,de,tn))) AND (('pregnancy'/exp OR 'pregnancy complication'/exp OR 'maternal care'/exp OR 'maternal exposure'/exp OR 'obstetrics'/exp OR 'obstetric procedure'/exp) OR (pregnant* OR obstetric* OR maternal* OR perinat* OR prenat*)))))) AND [embase]/lim

***************************

**[10]**

**Database: MEDLINE**

**Platform: Ovid**

**Date of Search: 2/15/22**

**# of Retrieved Results: 16**

**Search Strategy:**

1 ((screen* or survey* or question* or ask*) adj7 ((employ* or unemploy* or job or jobs or work or working or occupation* or career*) adj3 (status* or situation* or condition* or histor*))).mp. [mp=title, abstract, original title, name of substance word, subject heading word, floating sub-heading word, keyword heading word, organism supplementary concept word, protocol supplementary concept word, rare disease supplementary concept word, unique identifier, synonyms] (3100)

2 exp Mass Screening/ (138570)

3 exp Employment/ (94544)

4 2 and 3 (856)

5 AAFP Social Needs Screening Tool.mp. [mp=title, abstract, original title, name of substance word, subject heading word, floating sub-heading word, keyword heading word, organism supplementary concept word, protocol supplementary concept word, rare disease supplementary concept word, unique identifier, synonyms] (0)

6 Social Needs Screening Tool*.mp. [mp=title, abstract, original title, name of substance word, subject heading word, floating sub-heading word, keyword heading word, organism supplementary concept word, protocol supplementary concept word, rare disease supplementary concept word, unique identifier, synonyms] (8)

7 AccessHealth Spartanburg Screening Tool.mp. [mp=title, abstract, original title, name of substance word, subject heading word, floating sub-heading word, keyword heading word, organism supplementary concept word, protocol supplementary concept word, rare disease supplementary concept word, unique identifier, synonyms] (0)

8 ((AccessHealth or Spartanburg) adj3 Screening Tool*).mp. [mp=title, abstract, original title, name of substance word, subject heading word, floating sub-heading word, keyword heading word, organism supplementary concept word, protocol supplementary concept word, rare disease supplementary concept word, unique identifier, synonyms] (0)

9 Accountable Health Communities Health-Related Social Needs Screening Tool.mp. [mp=title, abstract, original title, name of substance word, subject heading word, floating sub-heading word, keyword heading word, organism supplementary concept word, protocol supplementary concept word, rare disease supplementary concept word, unique identifier, synonyms] (0)

10 Boston Medical Center-Thrive Screening Tool.mp. [mp=title, abstract, original title, name of substance word, subject heading word, floating sub-heading word, keyword heading word, organism supplementary concept word, protocol supplementary concept word, rare disease supplementary concept word, unique identifier, synonyms] (0)

11 HealthBegins Upstream Risk Screening Tool.mp. [mp=title, abstract, original title, name of substance word, subject heading word, floating sub-heading word, keyword heading word, organism supplementary concept word, protocol supplementary concept word, rare disease supplementary concept word, unique identifier, synonyms] (0)

12 Medical-Legal Partnership IHELLP.mp. [mp=title, abstract, original title, name of substance word, subject heading word, floating sub-heading word, keyword heading word, organism supplementary concept word, protocol supplementary concept word, rare disease supplementary concept word, unique identifier, synonyms] (0)

13 PRAPARE.mp. [mp=title, abstract, original title, name of substance word, subject heading word, floating sub-heading word, keyword heading word, organism supplementary concept word, protocol supplementary concept word, rare disease supplementary concept word, unique identifier, synonyms] (10)

14 WellRx Toolkit.mp. [mp=title, abstract, original title, name of substance word, subject heading word, floating sub-heading word, keyword heading word, organism supplementary concept word, protocol supplementary concept word, rare disease supplementary concept word, unique identifier, synonyms] (0)

15 We Care Screening Tool.mp. [mp=title, abstract, original title, name of substance word, subject heading word, floating sub-heading word, keyword heading word, organism supplementary concept word, protocol supplementary concept word, rare disease supplementary concept word, unique identifier, synonyms] (1)

16 iscreen.mp. [mp=title, abstract, original title, name of substance word, subject heading word, floating sub-heading word, keyword heading word, organism supplementary concept word, protocol supplementary concept word, rare disease supplementary concept word, unique identifier, synonyms] (16)

17 Family fIRST.mp. [mp=title, abstract, original title, name of substance word, subject heading word, floating sub-heading word, keyword heading word, organism supplementary concept word, protocol supplementary concept word, rare disease supplementary concept word, unique identifier, synonyms] (193)

18 screen*.mp. [mp=title, abstract, original title, name of substance word, subject heading word, floating sub-heading word, keyword heading word, organism supplementary concept word, protocol supplementary concept word, rare disease supplementary concept word, unique identifier, synonyms] (943223)

19 17 and 18 (9)

20 ((social* or psychosoc*) adj5 (determin* or risk*) adj7 (employment* or employed or employee* or unemploy* or job or jobs or occupation*)).mp. [mp=title, abstract, original title, name of substance word, subject heading word, floating sub-heading word, keyword heading word, organism supplementary concept word, protocol supplementary concept word, rare disease supplementary concept word, unique identifier, synonyms] (944)

21 exp "Social Determinants of Health"/ (5249)

22 ((social* or psychosoc*) adj2 (determin* or risk*) adj5 (health* or unhealth*)).mp. [mp=title, abstract, original title, name of substance word, subject heading word, floating sub-heading word, keyword heading word, organism supplementary concept word, protocol supplementary concept word, rare disease supplementary concept word, unique identifier, synonyms] (13082)

23 21 or 22 (13082)

24 exp Socioeconomic Factors/ (484731)

25 (employ* or unemploy* or job or jobs or work or worked or works or working).mp. [mp=title, abstract, original title, name of substance word, subject heading word, floating sub-heading word, keyword heading word, organism supplementary concept word, protocol supplementary concept word, rare disease supplementary concept word, unique identifier, synonyms] (2157439)

26 23 or 24 (493691)

27 2 and 25 and 26 (1385)

28 ((social* or psychosoc*) adj5 (determin* or risk*) adj7 (employ* or unemploy* or job or jobs or work or worked or works or working)).mp. (1633)

29 ((social* or psychosoc*) adj5 (determin* or risk*) adj7 ((employ* or unemploy* or job or jobs or work or worked or works or working) adj5 (question* or screen* or tool* or survey* or ask* or answer*))).mp. (52)

30 exp Data Collection/ (2409888)

31 exp Health Status/ (395171)

32 26 or 31 (836909)

33 30 and 32 (335452)

34 3 and 33 (27842)

35 2 and 34 (569)

36 (question* or screen* or tool* or survey* or ask* or answer*).mp. [mp=title, abstract, original title, name of substance word, subject heading word, floating sub-heading word, keyword heading word, organism supplementary concept word, protocol supplementary concept word, rare disease supplementary concept word, unique identifier, synonyms] (3466989)

37 28 and 36 (842)

38 1 or 4 or 29 or 35 or 37 (4755)

39 exp General Practice/ (77128)

40 general practitioners/ or physicians, family/ or physicians, primary care/ (30174)

41 exp Primary Health Care/ (179745)

42 39 or 40 or 41 (267469)

43 ((family adj3 (medic* or care or healthcare or practic*)) or (general adj3 (medic* or care or healthcare or practic*)) or (primary adj3 (care or healthcare))).mp. [mp=title, abstract, original title, name of substance word, subject heading word, floating sub-heading word, keyword heading word, organism supplementary concept word, protocol supplementary concept word, rare disease supplementary concept word, unique identifier, synonyms] (332578)

44 42 or 43 (429130)

45 38 and 44 (225)

46 5 or 6 or 7 or 8 or 9 or 10 or 11 or 12 or 13 or 14 or 15 or 16 or 17 (227)

47 44 and 46 (17)

48 45 or 47 (242)

49 20 or 23 (13784)

50 2 or 36 (3475089)

51 44 and 49 and 50 (576)

52 51 not 48 (554)

53 (202105* or 202106* or 202107* or 202108* or 202109* or 20211* or 2022*).ed. (1262466)

54 48 and 53 (16)

55 52 and 53 (108)

***************************

**[11]**

**Database: MEDLINE**

**Platform: Ovid**

**Date of Search: 2/15/22**

**# of Retrieved Results: 108**

**Search Strategy:**

1 ((screen* or survey* or question* or ask*) adj7 ((employ* or unemploy* or job or jobs or work or working or occupation* or career*) adj3 (status* or situation* or condition* or histor*))).mp. [mp=title, abstract, original title, name of substance word, subject heading word, floating sub-heading word, keyword heading word, organism supplementary concept word, protocol supplementary concept word, rare disease supplementary concept word, unique identifier, synonyms] (3100)

2 exp Mass Screening/ (138570)

3 exp Employment/ (94544)

4 2 and 3 (856)

5 AAFP Social Needs Screening Tool.mp. [mp=title, abstract, original title, name of substance word, subject heading word, floating sub-heading word, keyword heading word, organism supplementary concept word, protocol supplementary concept word, rare disease supplementary concept word, unique identifier, synonyms] (0)

6 Social Needs Screening Tool*.mp. [mp=title, abstract, original title, name of substance word, subject heading word, floating sub-heading word, keyword heading word, organism supplementary concept word, protocol supplementary concept word, rare disease supplementary concept word, unique identifier, synonyms] (8)

7 AccessHealth Spartanburg Screening Tool.mp. [mp=title, abstract, original title, name of substance word, subject heading word, floating sub-heading word, keyword heading word, organism supplementary concept word, protocol supplementary concept word, rare disease supplementary concept word, unique identifier, synonyms] (0)

8 ((AccessHealth or Spartanburg) adj3 Screening Tool*).mp. [mp=title, abstract, original title, name of substance word, subject heading word, floating sub-heading word, keyword heading word, organism supplementary concept word, protocol supplementary concept word, rare disease supplementary concept word, unique identifier, synonyms] (0)

9 Accountable Health Communities Health-Related Social Needs Screening Tool.mp. [mp=title, abstract, original title, name of substance word, subject heading word, floating sub-heading word, keyword heading word, organism supplementary concept word, protocol supplementary concept word, rare disease supplementary concept word, unique identifier, synonyms] (0)

10 Boston Medical Center-Thrive Screening Tool.mp. [mp=title, abstract, original title, name of substance word, subject heading word, floating sub-heading word, keyword heading word, organism supplementary concept word, protocol supplementary concept word, rare disease supplementary concept word, unique identifier, synonyms] (0)

11 HealthBegins Upstream Risk Screening Tool.mp. [mp=title, abstract, original title, name of substance word, subject heading word, floating sub-heading word, keyword heading word, organism supplementary concept word, protocol supplementary concept word, rare disease supplementary concept word, unique identifier, synonyms] (0)

12 Medical-Legal Partnership IHELLP.mp. [mp=title, abstract, original title, name of substance word, subject heading word, floating sub-heading word, keyword heading word, organism supplementary concept word, protocol supplementary concept word, rare disease supplementary concept word, unique identifier, synonyms] (0)

13 PRAPARE.mp. [mp=title, abstract, original title, name of substance word, subject heading word, floating sub-heading word, keyword heading word, organism supplementary concept word, protocol supplementary concept word, rare disease supplementary concept word, unique identifier, synonyms] (10)

14 WellRx Toolkit.mp. [mp=title, abstract, original title, name of substance word, subject heading word, floating sub-heading word, keyword heading word, organism supplementary concept word, protocol supplementary concept word, rare disease supplementary concept word, unique identifier, synonyms] (0)

15 We Care Screening Tool.mp. [mp=title, abstract, original title, name of substance word, subject heading word, floating sub-heading word, keyword heading word, organism supplementary concept word, protocol supplementary concept word, rare disease supplementary concept word, unique identifier, synonyms] (1)

16 iscreen.mp. [mp=title, abstract, original title, name of substance word, subject heading word, floating sub-heading word, keyword heading word, organism supplementary concept word, protocol supplementary concept word, rare disease supplementary concept word, unique identifier, synonyms] (16)

17 Family fIRST.mp. [mp=title, abstract, original title, name of substance word, subject heading word, floating sub-heading word, keyword heading word, organism supplementary concept word, protocol supplementary concept word, rare disease supplementary concept word, unique identifier, synonyms] (193)

18 screen*.mp. [mp=title, abstract, original title, name of substance word, subject heading word, floating sub-heading word, keyword heading word, organism supplementary concept word, protocol supplementary concept word, rare disease supplementary concept word, unique identifier, synonyms] (943223)

19 17 and 18 (9)

20 ((social* or psychosoc*) adj5 (determin* or risk*) adj7 (employment* or employed or employee* or unemploy* or job or jobs or occupation*)).mp. [mp=title, abstract, original title, name of substance word, subject heading word, floating sub-heading word, keyword heading word, organism supplementary concept word, protocol supplementary concept word, rare disease supplementary concept word, unique identifier, synonyms] (944)

21 exp "Social Determinants of Health"/ (5249)

22 ((social* or psychosoc*) adj2 (determin* or risk*) adj5 (health* or unhealth*)).mp. [mp=title, abstract, original title, name of substance word, subject heading word, floating sub-heading word, keyword heading word, organism supplementary concept word, protocol supplementary concept word, rare disease supplementary concept word, unique identifier, synonyms] (13082)

23 21 or 22 (13082)

24 exp Socioeconomic Factors/ (484731)

25 (employ* or unemploy* or job or jobs or work or worked or works or working).mp. [mp=title, abstract, original title, name of substance word, subject heading word, floating sub-heading word, keyword heading word, organism supplementary concept word, protocol supplementary concept word, rare disease supplementary concept word, unique identifier, synonyms] (2157439)

26 23 or 24 (493691)

27 2 and 25 and 26 (1385)

28 ((social* or psychosoc*) adj5 (determin* or risk*) adj7 (employ* or unemploy* or job or jobs or work or worked or works or working)).mp. (1633)

29 ((social* or psychosoc*) adj5 (determin* or risk*) adj7 ((employ* or unemploy* or job or jobs or work or worked or works or working) adj5 (question* or screen* or tool* or survey* or ask* or answer*))).mp. (52)

30 exp Data Collection/ (2409888)

31 exp Health Status/ (395171)

32 26 or 31 (836909)

33 30 and 32 (335452)

34 3 and 33 (27842)

35 2 and 34 (569)

36 (question* or screen* or tool* or survey* or ask* or answer*).mp. [mp=title, abstract, original title, name of substance word, subject heading word, floating sub-heading word, keyword heading word, organism supplementary concept word, protocol supplementary concept word, rare disease supplementary concept word, unique identifier, synonyms] (3466989)

37 28 and 36 (842)

38 1 or 4 or 29 or 35 or 37 (4755)

39 exp General Practice/ (77128)

40 general practitioners/ or physicians, family/ or physicians, primary care/ (30174)

41 exp Primary Health Care/ (179745)

42 39 or 40 or 41 (267469)

43 ((family adj3 (medic* or care or healthcare or practic*)) or (general adj3 (medic* or care or healthcare or practic*)) or (primary adj3 (care or healthcare))).mp. [mp=title, abstract, original title, name of substance word, subject heading word, floating sub-heading word, keyword heading word, organism supplementary concept word, protocol supplementary concept word, rare disease supplementary concept word, unique identifier, synonyms] (332578)

44 42 or 43 (429130)

45 38 and 44 (225)

46 5 or 6 or 7 or 8 or 9 or 10 or 11 or 12 or 13 or 14 or 15 or 16 or 17 (227)

47 44 and 46 (17)

48 45 or 47 (242)

49 20 or 23 (13784)

50 2 or 36 (3475089)

51 44 and 49 and 50 (576)

52 51 not 48 (554)

53 (202105* or 202106* or 202107* or 202108* or 202109* or 20211* or 2022*).ed. (1262466)

54 48 and 53 (16)

55 52 and 53 (108)

***************************

**[12]**

**Database: APA PsycInfo**

**Platform: Ovid**

**Date of Search: 2/15/22**

**# of Retrieved Results: 16**

**Search Strategy:**

1 ((screen* or survey* or question* or ask*) adj7 ((employ* or unemploy* or job or jobs or work or working or occupation* or career*) adj3 (status* or situation* or condition* or histor*))).mp. [mp=title, abstract, heading word, table of contents, key concepts, original title, tests & measures, mesh word] (1865)

2 exp Screening/ (35144)

3 exp Employment/ (29554)

4 2 and 3 (136)

5 AAFP Social Needs Screening Tool.mp. [mp=title, abstract, heading word, table of contents, key concepts, original title, tests & measures, mesh word] (0)

6 Social Needs Screening Tool*.mp. [mp=title, abstract, heading word, table of contents, key concepts, original title, tests & measures, mesh word] (0)

7 AccessHealth Spartanburg Screening Tool.mp. [mp=title, abstract, heading word, table of contents, key concepts, original title, tests & measures, mesh word] (0)

8 ((AccessHealth or Spartanburg) adj3 Screening Tool*).mp. [mp=title, abstract, heading word, table of contents, key concepts, original title, tests & measures, mesh word] (0)

9 Accountable Health Communities Health-Related Social Needs Screening Tool.mp. [mp=title, abstract, heading word, table of contents, key concepts, original title, tests & measures, mesh word] (0)

10 Boston Medical Center-Thrive Screening Tool.mp. [mp=title, abstract, heading word, table of contents, key concepts, original title, tests & measures, mesh word] (0)

11 HealthBegins Upstream Risk Screening Tool.mp. [mp=title, abstract, heading word, table of contents, key concepts, original title, tests & measures, mesh word] (0)

12 Medical-Legal Partnership IHELLP.mp. [mp=title, abstract, heading word, table of contents, key concepts, original title, tests & measures, mesh word] (0)

13 PRAPARE.mp. [mp=title, abstract, heading word, table of contents, key concepts, original title, tests & measures, mesh word] (3)

14 WellRx Toolkit.mp. [mp=title, abstract, heading word, table of contents, key concepts, original title, tests & measures, mesh word] (0)

15 We Care Screening Tool.mp. [mp=title, abstract, heading word, table of contents, key concepts, original title, tests & measures, mesh word] (2)

16 iscreen.mp. [mp=title, abstract, heading word, table of contents, key concepts, original title, tests & measures, mesh word] (2)

17 Family fIRST.mp. [mp=title, abstract, heading word, table of contents, key concepts, original title, tests & measures, mesh word] (119)

18 screen*.mp. [mp=title, abstract, heading word, table of contents, key concepts, original title, tests & measures, mesh word] (133598)

19 17 and 18 (2)

20 ((social* or psychosoc*) adj5 (determin* or risk*) adj7 (employment* or employed or employee* or unemploy* or job or jobs or occupation*)).mp. [mp=title, abstract, heading word, table of contents, key concepts, original title, tests & measures, mesh word] (734)

21 exp Socioeconomic Factors/ (90670)

22 exp sociocultural factors/ (126173)

23 21 or 22 (211027)

24 exp health disparities/ or exp health/ (376743)

25 23 and 24 (26265)

26 ((social* or psychosoc*) adj2 (determin* or risk*) adj5 (health* or unhealth*)).mp. [mp=title, abstract, heading word, table of contents, key concepts, original title, tests & measures, mesh word] (3917)

27 25 or 26 (29646)

28 exp Socioeconomic Factors/ (90670)

29 exp sociocultural factors/ (126173)

30 (employ* or unemploy* or job or jobs or work or worked or works or working).mp. [mp=title, abstract, heading word, table of contents, key concepts, original title, tests & measures, mesh word] (980221)

31 27 or 28 (105011)

32 2 and 30 and 31 (238)

33 ((social* or psychosoc*) adj5 (determin* or risk*) adj7 (employ* or unemploy* or job or jobs or work or worked or works or working)).mp. (1640)

34 ((social* or psychosoc*) adj5 (determin* or risk*) adj7 ((employ* or unemploy* or job or jobs or work or worked or works or working) adj5 (question* or screen* or tool* or survey* or ask* or answer*))).mp. (50)

35 exp Data Collection/ (75239)

36 exp Health Status/ (49920)

37 31 or 36 (152338)

38 35 and 37 (2048)

39 3 and 38 (386)

40 2 and 39 (4)

41 (question* or screen* or tool* or survey* or ask* or answer*).mp. [mp=title, abstract, heading word, table of contents, key concepts, original title, tests & measures, mesh word] (1361756)

42 33 and 41 (720)

43 1 or 4 or 34 or 40 or 42 (2680)

44 exp pregnancy/ (45403)

45 exp prenatal development/ (8122)

46 exp obstetrics/ (3139)

47 exp perinatal period/ (3430)

48 44 or 45 or 46 or 47 (55052)

49 (pregnan* or obstet* or childbear* or perinatal* or peri-natal* or prenatal* or pre-natal* or (family adj2 planning) or ((maternal* or mother*) adj3 (health* or welfar* or expos* or risk* or harm* or advers* or affect* or effect* or impact* or inhibit* or interrupt* or interfer*))).mp. [mp=title, abstract, heading word, table of contents, key concepts, original title, tests & measures, mesh word] (109976)

50 48 or 49 (121617)

51 43 and 50 (69)

52 5 or 6 or 7 or 8 or 9 or 10 or 11 or 12 or 13 or 14 or 15 or 16 or 17 (126)

53 50 and 52 (10)

54 51 or 53 (79)

55 20 or 27 (30234)

56 2 or 41 (1364967)

57 50 and 55 and 56 (759)

58 57 not 54 (741)

59 48 and 55 and 56 (279)

60 54 or 59 (352)

61 (202105* or 202106* or 202107* or 202108* or 202109* or 20211* or 2022*).up. (145886)

62 60 and 61 (16)

***************************

**[13]**

**Database: MEDLINE**

**Platform: Ovid**

**Date of Search: 2/15/22**

**# of Retrieved Results: 7**

**Search Strategy:**

1 ((screen* or survey* or question* or ask*) adj7 ((employ* or unemploy* or job or jobs or work or working or occupation* or career*) adj3 (status* or situation* or condition* or histor*))).mp. [mp=title, abstract, original title, name of substance word, subject heading word, floating sub-heading word, keyword heading word, organism supplementary concept word, protocol supplementary concept word, rare disease supplementary concept word, unique identifier, synonyms] (3100)

2 exp Mass Screening/ (138570)

3 exp Employment/ (94544)

4 2 and 3 (856)

5 AAFP Social Needs Screening Tool.mp. [mp=title, abstract, original title, name of substance word, subject heading word, floating sub-heading word, keyword heading word, organism supplementary concept word, protocol supplementary concept word, rare disease supplementary concept word, unique identifier, synonyms] (0)

6 Social Needs Screening Tool*.mp. [mp=title, abstract, original title, name of substance word, subject heading word, floating sub-heading word, keyword heading word, organism supplementary concept word, protocol supplementary concept word, rare disease supplementary concept word, unique identifier, synonyms] (8)

7 AccessHealth Spartanburg Screening Tool.mp. [mp=title, abstract, original title, name of substance word, subject heading word, floating sub-heading word, keyword heading word, organism supplementary concept word, protocol supplementary concept word, rare disease supplementary concept word, unique identifier, synonyms] (0)

8 ((AccessHealth or Spartanburg) adj3 Screening Tool*).mp. [mp=title, abstract, original title, name of substance word, subject heading word, floating sub-heading word, keyword heading word, organism supplementary concept word, protocol supplementary concept word, rare disease supplementary concept word, unique identifier, synonyms] (0)

9 Accountable Health Communities Health-Related Social Needs Screening Tool.mp. [mp=title, abstract, original title, name of substance word, subject heading word, floating sub-heading word, keyword heading word, organism supplementary concept word, protocol supplementary concept word, rare disease supplementary concept word, unique identifier, synonyms] (0)

10 Boston Medical Center-Thrive Screening Tool.mp. [mp=title, abstract, original title, name of substance word, subject heading word, floating sub-heading word, keyword heading word, organism supplementary concept word, protocol supplementary concept word, rare disease supplementary concept word, unique identifier, synonyms] (0)

11 HealthBegins Upstream Risk Screening Tool.mp. [mp=title, abstract, original title, name of substance word, subject heading word, floating sub-heading word, keyword heading word, organism supplementary concept word, protocol supplementary concept word, rare disease supplementary concept word, unique identifier, synonyms] (0)

12 Medical-Legal Partnership IHELLP.mp. [mp=title, abstract, original title, name of substance word, subject heading word, floating sub-heading word, keyword heading word, organism supplementary concept word, protocol supplementary concept word, rare disease supplementary concept word, unique identifier, synonyms] (0)

13 PRAPARE.mp. [mp=title, abstract, original title, name of substance word, subject heading word, floating sub-heading word, keyword heading word, organism supplementary concept word, protocol supplementary concept word, rare disease supplementary concept word, unique identifier, synonyms] (10)

14 WellRx Toolkit.mp. [mp=title, abstract, original title, name of substance word, subject heading word, floating sub-heading word, keyword heading word, organism supplementary concept word, protocol supplementary concept word, rare disease supplementary concept word, unique identifier, synonyms] (0)

15 We Care Screening Tool.mp. [mp=title, abstract, original title, name of substance word, subject heading word, floating sub-heading word, keyword heading word, organism supplementary concept word, protocol supplementary concept word, rare disease supplementary concept word, unique identifier, synonyms] (1)

16 iscreen.mp. [mp=title, abstract, original title, name of substance word, subject heading word, floating sub-heading word, keyword heading word, organism supplementary concept word, protocol supplementary concept word, rare disease supplementary concept word, unique identifier, synonyms] (16)

17 Family fIRST.mp. [mp=title, abstract, original title, name of substance word, subject heading word, floating sub-heading word, keyword heading word, organism supplementary concept word, protocol supplementary concept word, rare disease supplementary concept word, unique identifier, synonyms] (193)

18 screen*.mp. [mp=title, abstract, original title, name of substance word, subject heading word, floating sub-heading word, keyword heading word, organism supplementary concept word, protocol supplementary concept word, rare disease supplementary concept word, unique identifier, synonyms] (943223)

19 17 and 18 (9)

20 ((social* or psychosoc*) adj5 (determin* or risk*) adj7 (employment* or employed or employee* or unemploy* or job or jobs or occupation*)).mp. [mp=title, abstract, original title, name of substance word, subject heading word, floating sub-heading word, keyword heading word, organism supplementary concept word, protocol supplementary concept word, rare disease supplementary concept word, unique identifier, synonyms] (944)

21 exp "Social Determinants of Health"/ (5249)

22 ((social* or psychosoc*) adj2 (determin* or risk*) adj5 (health* or unhealth*)).mp. [mp=title, abstract, original title, name of substance word, subject heading word, floating sub-heading word, keyword heading word, organism supplementary concept word, protocol supplementary concept word, rare disease supplementary concept word, unique identifier, synonyms] (13082)

23 21 or 22 (13082)

24 exp Socioeconomic Factors/ (484731)

25 (employ* or unemploy* or job or jobs or work or worked or works or working).mp. [mp=title, abstract, original title, name of substance word, subject heading word, floating sub-heading word, keyword heading word, organism supplementary concept word, protocol supplementary concept word, rare disease supplementary concept word, unique identifier, synonyms] (2157439)

26 23 or 24 (493691)

27 2 and 25 and 26 (1385)

28 ((social* or psychosoc*) adj5 (determin* or risk*) adj7 (employ* or unemploy* or job or jobs or work or worked or works or working)).mp. (1633)

29 ((social* or psychosoc*) adj5 (determin* or risk*) adj7 ((employ* or unemploy* or job or jobs or work or worked or works or working) adj5 (question* or screen* or tool* or survey* or ask* or answer*))).mp. (52)

30 exp Data Collection/ (2409888)

31 exp Health Status/ (395171)

32 26 or 31 (836909)

33 30 and 32 (335452)

34 3 and 33 (27842)

35 2 and 34 (569)

36 (question* or screen* or tool* or survey* or ask* or answer*).mp. [mp=title, abstract, original title, name of substance word, subject heading word, floating sub-heading word, keyword heading word, organism supplementary concept word, protocol supplementary concept word, rare disease supplementary concept word, unique identifier, synonyms] (3466989)

37 28 and 36 (842)

38 1 or 4 or 29 or 35 or 37 (4755)

39 exp General Practice/ (77128)

40 general practitioners/ or physicians, family/ or physicians, primary care/ (30174)

41 exp Primary Health Care/ (179745)

42 39 or 40 or 41 (267469)

43 ((family adj3 (medic* or care or healthcare or practic*)) or (general adj3 (medic* or care or healthcare or practic*)) or (primary adj3 (care or healthcare))).mp. [mp=title, abstract, original title, name of substance word, subject heading word, floating sub-heading word, keyword heading word, organism supplementary concept word, protocol supplementary concept word, rare disease supplementary concept word, unique identifier, synonyms] (332578)

44 42 or 43 (429130)

45 38 and 44 (225)

46 5 or 6 or 7 or 8 or 9 or 10 or 11 or 12 or 13 or 14 or 15 or 16 or 17 (227)

47 44 and 46 (17)

48 45 or 47 (242)

49 20 or 23 (13784)

50 2 or 36 (3475089)

51 44 and 49 and 50 (576)

52 51 not 48 (554)

53 4 and 33 (569)

54 3 and 23 and 30 (169)

55 53 or 54 (736)

56 55 not (48 or 51) (691)

57 exp "Delivery of Health Care"/ (1169645)

58 56 and 57 (146)

59 exp risk/ (1324864)

60 56 and 59 (176)

61 58 or 60 (281)

62 (202105* or 202106* or 202107* or 202108* or 202109* or 20211* or 2022*).ed. (1262466)

63 61 and 62 (7)

***************************
